# The ABCT31 Transporter Regulates the Export System of Phenylacetic Acid as a Side-Chain Precursor of Penicillin G in *Monascus ruber* M7

**DOI:** 10.3389/fmicb.2022.915721

**Published:** 2022-07-28

**Authors:** Rabia Ramzan, Muhammad Safiullah Virk, Fusheng Chen

**Affiliations:** ^1^Hubei International Scientific and Technological Cooperation Base of Traditional Fermented Foods, Huazhong Agricultural University, Wuhan, China; ^2^College of Food Science and Technology, Huazhong Agricultural University, Wuhan, China; ^3^Department of Food Science and Technology, Government College Women University, Faisalabad, Pakistan

**Keywords:** ABC transporter, secondary metabolites, phenylacetic acid, amino acid, weak acid

## Abstract

The biosynthesis of penicillin G (PG) is compartmentalized, and the transportation of the end and intermediate products, and substrates (precursors) such as L-cysteine (L-Cys), L-valine (L-Val) and phenylacetic acid (PAA) requires traversing membrane barriers. However, the transportation system of PAA as a side chain of PG are unclear yet. To discover ABC transporters (ABCTs) involved in the transportation of PAA, the expression levels of 38 ABCT genes in the genome of *Monascus ruber* M7, culturing with and without PAA, were examined, and found that one *abct* gene, namely *abct*31, was considerably up-regulated with PAA, indicating that *abct*31 may be relative with PAA transportation. Furthermore the disruption of *abct*31 was carried out, and the effects of two PG substrate's amino acids (L-Cys and L-Val), PAA and some other weak acids on the morphologies and production of secondary metabolites (SMs) of Δ*abct*31 and *M. ruber* M7, were performed through feeding experiments. The results revealed that L-Cys, L-Val and PAA substantially impacted the morphologies and SMs production of Δ*abct*31 and *M. ruber* M7. The UPLC-MS/MS analysis findings demonstrated that Δ*abct*31 did not interrupt the synthesis of PG in *M. ruber* M7. According to the results, it suggests that *abct*31 is involved in the resistance and detoxification of the weak acids, including the PAA in *M. ruber* M7.

## Introduction

In view of the present antibiotic resistance crises and the slowing rate of antibiotic discovery, there has been a worldwide push to look into the large quantity of unexplored natural product sources for novel antibiotics or their precursors (Seiple et al., 2016). Even though β-lactam antibiotics have been present since the 1920s, they remained the most commonly utilized antibiotic class. Meanwhile, new routes for biosynthesis of β-lactam are continually being found (Gaudelli et al., 2015). In China and some other Asian regions, *Monascus* spp. are popular as conventional edible fungi. Their fermented products, namely red yeast rice, red mold rice (RMR), Anka, or Hongqu, have been widely utilized in China as folk cures and culinary colorants for 2,000 years (Lee et al., 2010; Lee and Pan, 2012; Guo et al., 2019). Researchers have publicized that *Monascus* spp. can create both useful and harmful secondary metabolites (SMs), such as *Monascus* pigments, aminobutyric acid, dimurumic acid, and monacolin K, and a typical type of mycotoxin, which is known as citrinin (Ozcengiz and Demain, 2013; Yuliana et al., 2017; Chen and Pan, 2019). Recently, Chen (2015) found a probable gene cluster having liability for β-lactam synthesis in the genome of *M. ruber* M7, and one β-lactam biosynthesis gene was identified in the gene cluster (Ramzan et al., 2019). *M. purpureus* was also proved to produce γ-lactam according to Wei et al. (2017).

Many microorganisms, including filamentous fungi, actinomycetes, and Gram-negative bacteria, may synthesize several types of β-lactam antibiotics (Lobanovska and Pilla, 2017). During the biosynthetic pathway of the β-lactam antibiotics, the non-ribosomal peptide synthetase initially condenses the three precursor amino acids, L-α-aminoadipic acid, L-cysteine and L-valine, to form the basic nucleus of β-lactam antibiotic, isopenicillin N (IPN) with hydrophilic feature (Fernández-Aguado et al., 2014). The IPN fabrication is taken place in the cytoplasm of β-lactam producers (Barreiro and García-Estrada, 2019). Then, the conversion of IPN is taken place into penicillin G (PG) in the peroxisomal matrix of β-lactam producers (Van Den Berg et al., 2008; Ávalos et al., 2014). During this process, an hydrophilic α-aminoadipyl side chain, phenylacetic acid (PAA), is catalyzed into a hydrophobic phenyl acetyl CoA of IPN by the IPN acyl transferase (García-Estrada et al., 2008). The entry of the side chain of PG, PAA, into the cells takes place via passive diffusion. And acidification of cytosol is caused by the influx of PAA. After entering the cells, PAA is dissociated and releases protons. Due to the acidification of cytoplasm, enzymatic activities are inhibited, which causes the ultimate death of the cell (Weber et al., 2012). Nevertheless, cytoplasmic acidification is unlikely to be the only cause of mild acid toxicity. The more hydrophobic sorbic acid, for example, may impair the organization of the membrane, leading to oxidative stress due to a lesser functioning of the respiratory chain, and so toxicity could be a multidimensional phenomenon. So, during the biosynthesis of penicillin, the shipping of intermediates and their precursors across the cellular membranes is an important matter (Martín et al., 2005). Several transporters are not fully explored (Fernández-Aguado et al., 2013, 2014). According to their abilities to transport diverse organic composites, the transporters are classified into the extrusion of multidrug and toxic compounds, small multidrug resistance, ATP-binding cassette (ABC) superfamily, major facilitator superfamily, and so on (Martín et al., 2005).

Binding and hydrolyzing ATP to transport the substrates across the lipid bilayer is the characteristic ability of the ABC transporter family (Wilkens, 2015). The well-studied ABC exporters in prokaryotes and eukaryotes play a central role in life activities. There have been expansive investigations of their association with many human diseases (Fletcher and Mullins, 2010; Tang et al., 2021) and resistance to multiple drugs (Al Shawi et al., 2011). Weber et al. (2012) found that PAA in higher concentrations could be toxic to the cells, and the proliferation of the common ancestor of the recent penicillin-producing strains, *P. chrysogenum* Wisconsin 54-1255, is not possible at a high concentration (18 mM) of PAA. The high penicillin-producing strain *P. chrysogenum* DS17690, on the other hand, is generally insensitive to such PAA doses, showing that DS17690 cells have established PAA resistance mechanisms. Likewise, a weak acid extrusion system has been identified in *Saccharomyces cerevisiae*. PDR12 is an ABC transporter from *S. cerevisiae* that prevents cytoplasmic acidification and transports metabolites of yeast metabolism out of the cells (Hazelwood et al., 2006; Zhang et al., 2021).

Though several features of the traffic systems of β-lactam antibiotics need to be investigated further, it has been thought that the involvement of the transmembrane transporter proteins plays a role in their synthesis. As a result, more researches about the involvement of transporters in the β-lactam antibiotic gene cluster are required. Moreover, it has been strongly predicted that the ABC transporters encoding genes are present in the gene clusters. In the present study, the expression and identification of all ABC transporters present in the genome of *M. ruber* M7 for the biosynthesis of the β-lactam antibiotics have been investigated after being grown in the presence and absence of PAA. In the presence of PAA, the results revealed one ABC transporter (ABCT31), whose role in β-lactam antibiotic production was explored via *abct31*-deletion. Furthermore, compared to *M. ruber* M7, the resistance of the mutant to the weak acid toxicity was assessed at various concentrations with the presence and absence of PAA.

## Materials and Methods

### Materials

The Δ*abct*31 mutant was created using the *M. ruber* M7 strain (CCAM 070120, Culture Collection of State Key Laboratory of Agricultural Microbiology, < city>Wuhan < /city>, China), which can produce *Monascus* pigments and citrinin but not monacolin K. (Chen and Hu, 2005). Czapek yeast extract agar (CYA), potato dextrose agar (PDA), glycerol nitrate agar (G25N 25%), and malt extract agar (MA) media were used for the phenotypic analyses (He et al., 2013). On PDA, resistance markers hygromycin B (30 μg/mL) and neomycin (15 μg/mL) were used for transformants screening. PDA slants of strains were prepared and kept at 28°C.

### Extraction of the DNA

The strains were cultured on the PDA plates with cellophane covers. Extraction of the genomic DNA of the grown strains from their mycelia was done using the cetyltrimethylammonium bromide method by Shao et al. (2009).

### Quantitative Real-Time PCR (qRT-PCR) Analysis

The method described by Liu et al. (2014) was followed to conduct the qRT-PCR using the SLAN Fluorescence Quantitative Detection System from Wuhan Good Biotechnology Co., Ltd., (Wuhan, Hubei, China).

### Computational Structural Analysis of *abct*31

Table 1 lists the primer pairs utilized in the current investigation. The *abct*31gene was amplified by PCR using *M. ruber* M7 genomic DNA under the subsequent conditions. The first denaturation step was performed for 5 min at 94°C, preceded by 35 amplification cycles at 94°C for 30 s, at 58°C for 30 s, and for 1 min at 72°C. However, the T100 Thermal Cycler was used at the end during the extension phase at 72°C for 10 min (Bio-Rad, Hercules, CA, USA) (Chen et al., 2012a,b). SoftBerry's FGENESH software (https://linuxl.softberry.com/berry.phtml) was used to anticipate the ABCT31 amino acid sequences. The Pfam 27.0 program (http://pfam.xfam.org/) was used to examine the ABCT31 functional regions. While the BLASTP program (https://blast.ncbi.nlm.nih.gov/Blast.cgi) was utilized to interpret the homology of the presumed amino acid sequences of ABCT31. Moreover, the PSIPREDv3.2 server (http://bioi nf.cs.ucl.ac.uk/psip red/) (Buchan et al., 2013; Waterhouse et al., 2018) and RaptorX server (http://rapt orx.uchi cago.edu/) (Wang et al., 2016b) were employed, respectively, to explain the predictions of genes for secondary structural elements. Similarly, from the PSIPRED server, HMMTOP, MEMSAT3, and MEMSAT-SVM programs were used to describe membrane helix and topology predictions (Pandey et al., 2018). The Deep Loc-1.0 server (https://services.healthtech.dtu.dk/service.php?DeepLoc-1.0) was used to determine the likely subcellular protein localization (Emanuelsson et al., 2000). Predictions of all genes ontology domains for the probable gene term annotations were done using the FFPred 3 program (http://bioinf.cs.ucl.ac.uk/psipred/) (Pandey et al., 2018).

**Table 1:** Primers for all ABC transporter genes in *M. ruber* M7 and used in the construction of deletion cassettes for the gene *abct*31.

**Name**	**Sequence 5' 3'**	**Product length**
AbctF1	ACAAAAGCCAAAGGATGACGA	193
AbctR1	CGTCTGTTACTGCTGAACGCTA	
AbctF2	TCCTCGGGTTCATTTTCGTC	175
AbctR2	TTCACCCCAGCTGTACCAA	
AbctF3	ACGCTCACGAATCATAAGTGGA	193
AbctR3	CTGCCATGACCTCTCCGTTC	
AbctF4	ACTAACAGGAGCTAAGCCCAA	183
AbctR4	GCTGCACATGTATTATCTTGACC	
AbctF5	GCATATACCTACGCAGTGTCC	191
AbctR5	GCCAATACCTCGACCGCTA	
AbctF6	CCTCTGAATTTGCAATGCCTT	151
AbctR6	ACCCCATCTCTTCAAAATATGCT	
AbctF7	CTCAAGATAAAGCAGCAATCCAC	155
AbctR7	ATCGGTTTGAGGAGGGTTCTA	
AbctF8	AGATCAGCATTAAGCGCCACA	168
AbctR8	CATGTCCTTAATTATCGCCAGT	
AbctF9	TCCTGCTTTGTACATCGTCCA	175
AbctR9	TATACTGTAACCGCGCTAGCC	
AbctF10	GGCGTACTTCAAACAGACCGAT	176
AbctR10	TCGTCTTCTCCTCGGGCTCT	
AbctF11	ATGATCCCTGTCAGTGCTCCC	158
AbctR11	TCGTCCTTTTCCTCCGCACAC	
AbctF12	CCCAATGACCATATACACGAA	173
AbctR12	TAGACGACAAGACGCTCCA	
AbctF13	ACATGACAATGCCTGAAAGGTT	188
AbctR13	CCAACGGCCAGCATACCTC	
AbctF14	CAAAAGTACCCTGCTAGACCA	200
AbctR14	GGTTTGATTTGCAAGCTCCT	
AbctF15	GAGTCTGCACCAACATGATCG	176
AbctR15	ATATGTACCAGCAGACGCAAT	
AbctF16	AGACAGCCAGTTCTACGCATC	191
AbctR16	TTGAGATAGAGCCCGCCAT	
AbctF17	ATGCCGTCAATAGCTCGACA	152
AbctR17	ATTCAGGACCTATGGCCTT	
AbctF18	CACCAACTCTCCGCTCAGT	198
AbctR18	CTGTTTTGCCTCCCGTCT	
AbctF19	TTATTGTGCTCGCCTTCGTTG	174
AbctR19	TTCTTCAGCCCGACCGTTG	
AbctF20	GTTTCTCTAATTCTGGGACCG	93
AbctR20	ATATATGCTGGAAGTTGGCTC	
AbctF21	CACTTTTGCACACCAATCCAG	173
AbctR21	AAAGAATAACCAGACCGCACT	
AbctF22	ACGTCGAAGAGCAGAGGGATG	85
AbctR22	AGCATCTGGAGCCGTCACT	
AbctF23	CCAAACACACTAAGCCCGAAC	194
AbctR23	ATAAGCATCACCACGTAGCAAG	
AbctF24	TTCAGCAGCAAGATCTACACT	143
AbctR24	CTCCATGTCAAGAACCTCGAT	
AbctF25	CCCGTATGTCATAGTAGGCTT	177
AbctR25	CATGGCCTTCAGACTAGCAG	
AbctF26	AACGCCATAACCTCCATTGCT	151
AbctR26	CCATCAACCGCACAACTCCC	
AbctF27	AATCGCATCAATTAGTCTGGG	146
AbctR27	CATTCGTCCAGGTATGCAAC	
AbctF28	TCAATTTCTGGACGCCGTGA	190
AbctR28	AGGCTCAAAACTTCAACGTCT	
AbctF29	TGAAATCCAGGTCTCACGGAA	162
AbctR29	TGTTGTACACGGGCAATCCTC	
AbctF30	GCCAGCGTCCATCACAAAA	160
AbctR30	ACTCCATTACAAACTGGCCGACT	
AbctF31	TCCCAGCCAAATTTTCCGTTC	119
AbctR31	CAAGATTGCAAAAGCTCGAGT	
AbctF32	ACTACACGATTTCTGAAGCCTA	141
AbctR32	AGAACAGTCACAATACGTCCA	
AbctF33	TTGCCATGTCTGTGAATACCG	164
AbctR33	TCTTGGAAGCCTTAGTCCGTA	
AbctF34	CAGTACAAAGCACGCCACA	176
AbctR34	AGCAGCTACACAATACTGGTC	
AbctF35	GAAGCCTACCAAGTCCCCT	177
AbctR35	ATACAGAACCCGATGTCCAC	
AbctF36	ACTATAATGACGAGCGCCACT	153
AbctR36	CTTGAACAAATTCTTCGCACAGG	
AbctF37	GGCATCTATCCTGGCAACCC	170
AbctR37	TGACAACTTTATTCCCTGCGGTA	
AbctF38	TCGATGTCAATAAAGCGCAGT	127
AbctR38	AGGCGATTATTCTCTTTGTCGT	
γ-actin F	CTGGCGGTATCCACGTCACC	
γ-actin R	AGGCCAGAATGGATCCACCG	
*abct*31-5F *abct*31-5R	CCCAAGCTTCAAGGGCTGTGCT CGGTA CAATATCATCTTCTGTCGACTGA AAACAGCGGCAATGG	For the amplification of the 770 bp of the 5' flanking region of the *abct*31 gene
*abct*31-3F *abct*31-3R	GAGGTAATCCTTCTTTCTAGGTCA TCCTCGGCTTTTGC CGGGGTACCTGTGCGGT GAATAGGTGC	For the amplification of the 434 bp of the 3' flanking region of the *abct*31 gene
hphF hphR	GTCGACAGAAGATGATATTG CTAGAAAGAAGGATTACCTC	For the amplification of the 2,317 bp of the hph cassette from the plasmid pSKH
*abct*31F *abct*31R	CGCAATGGAGAAGACGGT CGGTGCGATTGAGTTCCA	For the amplification of the 750 bp of the partial *abct*31 gene

### Construction of the *abct*31 Gene Deletion

**Figure 1:**
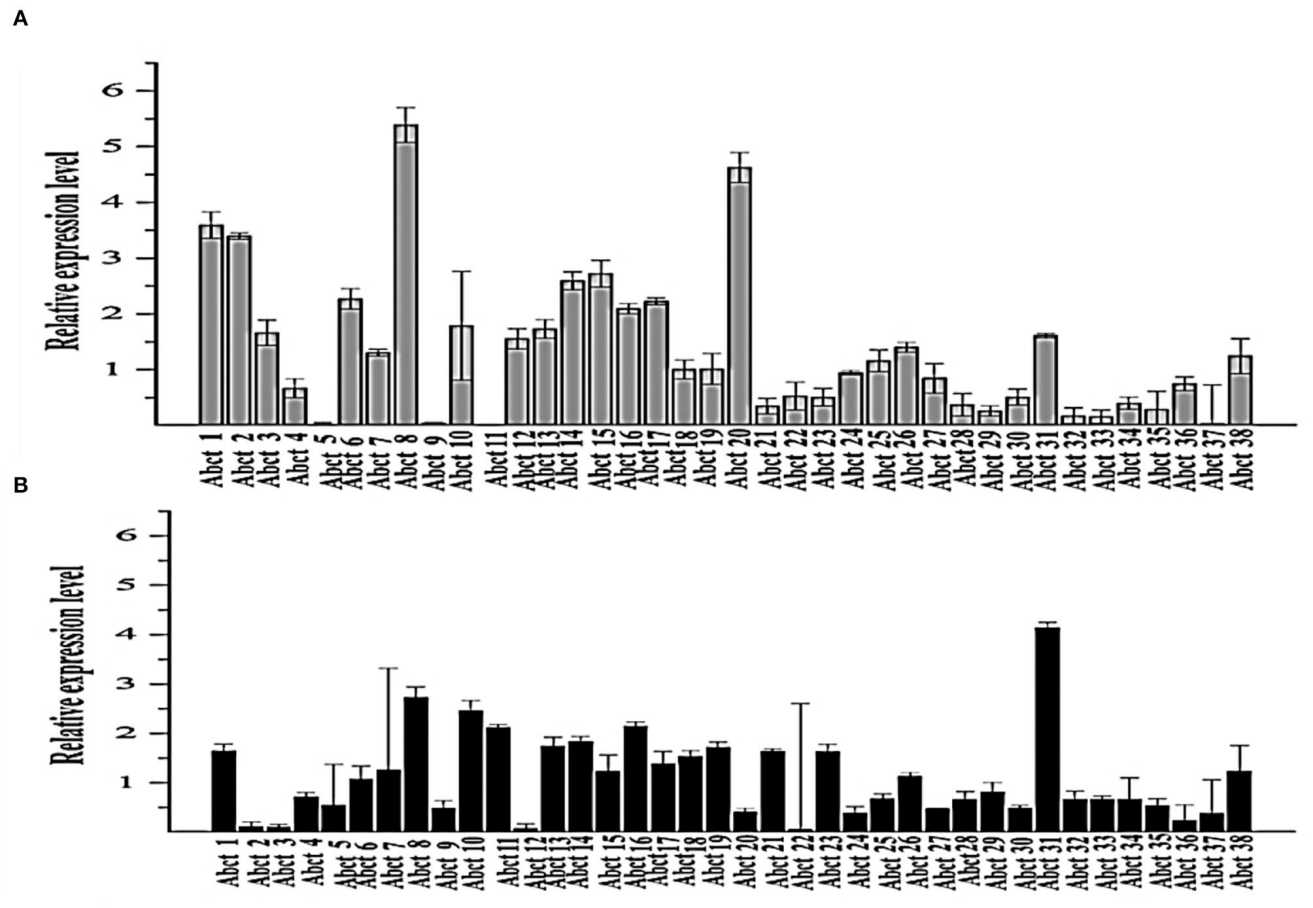
Quantitative real-time PCR analysis of the expression of all ABC transporter genes in the *M. ruber* M7 toward PAA. **(A)** The comparative expression of all ABC transporter genes in *M. ruber* M7 without PAA; **(B)** A comparative expression of all ABC transporter genes in *M. ruber* M7 with PAA. *M. ruber* M7 was cultivated and incubated at 28°C for 7 d in the presence (black bar) and absence (gray bar) of PAA at 10 mM. The values were represented as average values ± sd.

The approach of Shao et al. (2009) was used to delete the targeted gene *abct*31. The construction of the cassette for gene disruption (5'UTR-*hph*-3'UTR) was carried out with a double-joint PCR procedure. The primer pairs are listed in Table 1, whereas Figure 1A demonstrates the schematic illustration (Wang et al., 2011). For the development of the deletion strain (Δ*abct*31), construction of the *Agrobacterium tumefaciens* cells expressing the disruption vector (pC- *abct*31) of *abct*31 was done, and its co-cultivation was carried out with *M. ruber* M7.

### Southern Hybridization Analysis

The *abct*31 gene deletion strain was further confirmed utilizing the DIG-High Prime DNA Labeling & Detection Starter kit's method by applying PCR and Southern blot method (Roche, Mannheim, Germany). The restriction enzyme XbaI was used to digest the DNA of the putative Δ*abct*31 strain and *M. ruber* M7, respectively. As shown in Table 1, the respective primer pairs hphF/hphR and *abct*31F/*abct*31R were used to amplify the *hph* gene (probe 2) and *abct*31 gene (probe 1) by the PCR. Finally, probes 1 and 2 confirmed the Δ*abct*31 strain.

### Phenotypic Characterization

In order to study the phenotypic characteristics, colonial and microbiological features of parental (*M. ruber* M7) and Δ*abct*31strains were inoculated on PDA, G25N, MA, and CYA media plates and incubated at 28°C for 15 days (Chen et al., 2017a).

### Estimation of the Biomass

Estimation of the biomasses of *M. ruber* M7 and Δ*abct*31 was accomplished by the gravimetric method. The collection of mycelia on PDA plates was done following the drying at 60°C until the constant weights were obtained. Three replicates were used to compute the mean biomasses (Wang et al., 2016a).

### PG Contents' Extraction and Measurement

#### PG Extraction

The PG was extracted from the respective *M. ruber* M7 (wild-type) and Δ*abct*31 by the previously defined method (Ramzan et al., 2019).

#### HPLC Detection of the PG

The previously reported method (Ullán et al., 2008; Ramzan et al., 2019) was used for the analysis of intra- and extracellular PG contents.

#### Verification of PG and IPN by UPLC-MS/MS

The mass profile of the extract was produced using electrospray ionization (ESI) coupled Acquity TQD tandem quadruple mass spectrometer (Waters, Manchester, UK). The conditions for the ESI-MS to detect MS were set according to Ramzan et al. (2019). The presence of PG metabolites in the extract was confirmed using a PG standard with a purity of more than 98.0 percent (Sigma-Aldrich) (Lopes et al., 2013).

### Effects of Precursor Amino Acids Feeding on PG Production

The feeding of amino acids was used to investigate the transportation role of the *abct*31 gene (Yang et al., 2012). For this purpose, Δ*abct*31 and *M. ruber* M7 were separately grown on PDA with 2 mM concentrations of PG pathway amino acids such as D-Val, L-Cys and PAA, respectively. Then, phenotypic characteristics, biomasses, and PG synthesis were analyzed.

### Weak Acids Sensitivity Experiments

The feeding of mutants and *M. ruber* M7 on PDA with the addition of some weak acids including PAA, acetic acid, adipic acid and sorbic acid was used to examine the transportation mechanism of the *abct*31 gene (Weber et al., 2012). This procedure was carried out according to the method described by Weber et al. (2012) with slight modification such as PAA (mM): 50, 62.5, 75, 100, and 125; acetic acid (mM): 5, 10, 15, 20, and 25; adipic acid (mM): 12.5, 25, 37.5, and 50; and sorbic acid (mM): 2, 2.75, 3.5, and 4.25 concentration. The phenotypical features and relative expression of *abct*31 gene in *M. ruber* M7 were analyzed.

### Statistical Analyses

All of the experiments were carried out in triplicate. The statistical studies were performed using the Statistics 8.1 program (Analytical Software, USA). After performing the Tukey test, a *P* < 0.05 was taken as statistically significant, and a *P* < 0.01 was considered highly significant.

## Results and Discussion

### Evaluation of ABCT31 Sensitivity Toward PAA

To understand the proper mechanism of the *abct*31 gene as a transporter in the translocation of intermediates metabolites of the PG pathway, *M. ruber* M7 was cultivated for 7 days at 10 mM in the +PAA (presence) or –PAA (absence). The determination of the transcript levels of all ABC transporter genes in the genome of *M. ruber* M7 was done while the γ-actin gene used as a reference gene (Figure 1).

The ABC transporter expression levels have been noted in *M. ruber* M7 with or without PAA. The experiment results revealed a notable upsurge in the expression of *abct*31 (Figure 1B) in the presence of PAA in *M. ruber* M7, whereas without the PAA (Figure 1A), the expression of *abct*31 was considerably lower. In the +PAA/–PAA, all other ABC transporter genes displayed no expression or did not show a distinct variation in their transcript levels in *M. ruber* M7 (Figures 1A,B).

### Identification of Putative Function of *abct*31

**Figure 2:**
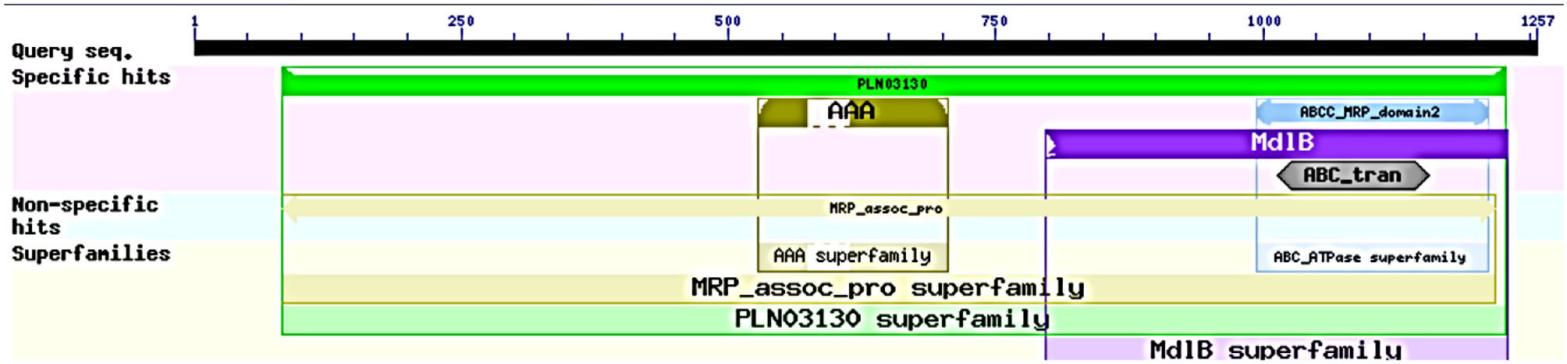
Domain analysis of *abct*31 analyzed by CDD program.

The ABCT31 protein sequence from *M. ruber* M7 was analyzed using NCBI's blast tool (https://blast.ncbi.nlm.nih.gov/Blast.cgi) and homologous aligning of the *abct*31 with 27 distinct ABC transporters from other genomes as well as identification of different conserved domains were discovered (Table 2). Softberry's FGENESH software was also used to predict the 1,257 amino acid sequence. *abct*31 corresponds to the ABC transporter family, according to a database search using the Pfam 27.0 tool (http://pfam.xfam.org/). The expression of an ABC-membrane family C domain (PLN03130, pfam00005) represents hydrophobic transmembrane helices, (Schulz and Kolukisaoglu, 2006), and a key component of ABC transport proteins and its recognition is conserved by the low E values (Figure 2).

**Table 2:** Putative functions of ABCT31 analyzed by Pblast and CDD program.

**Name**	**Accession**	**Description**	**Interval**
MRP-assoc-pro	TIGR00957	Mutltidrug resistance protein	29–1017
PLN03130	PLN03130	ABC transporter C family member	29–1027
ABC_tran	pfam00005	ABC transporter family responsible for translocation	722–955

### Model Validation and Homology Modeling

**Figure 3:**
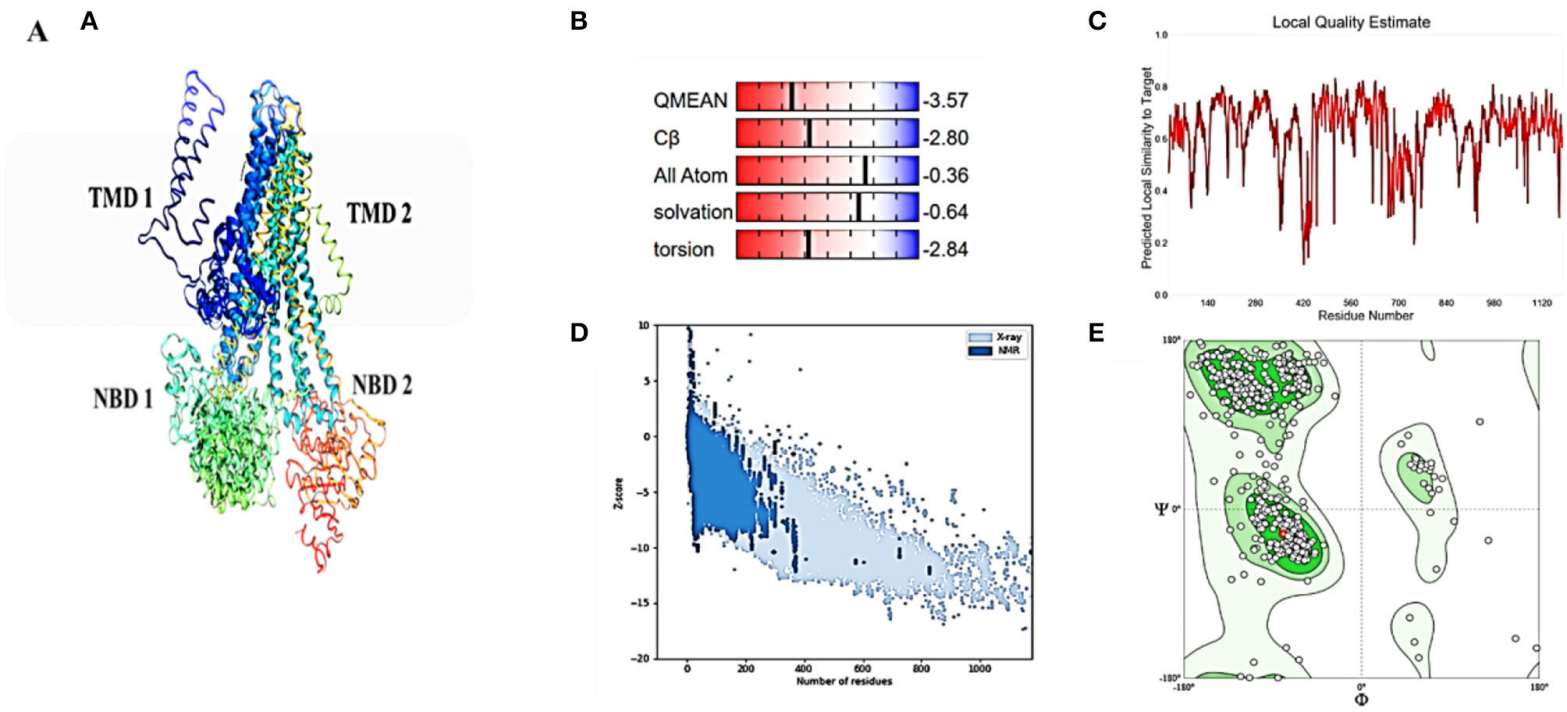
Homology model and checking results for ABCT31 protein. Verification of homology modeling **(A)** Homology model of ABCT31 protein TMD, Transmembrane domain; NBD, Nucleotide-binding domains; **(B)** Plot displaying the model's normalized Q mean score; **(C)** Plot displaying the model's local quality; **(D)** Z score value of the model developed; **(E)** Ramachandran plot validating the energy-minimized model's backbone dihedral angles.

Although atomic resolution crystal structures of various soluble proteins have been discovered, equivalent advancement has not been reported for transporter proteins. The key challenge is the severe exertion in crystallizing them due to flexibility in their conformation. As a result, developing a homology-based protein model is a viable option (Gao et al., 2021). The ABCT31's final 3D structure (Figure 3A), which was created using the SWISS-MODEL software and a template-based homology model, was visualized using Procheck. The basic layout of all ABC transporters is the same, with two transmembrane domains (TMDs) and two nucleotide-binding domains (NBDs). The structural analysis has revealed that ABCT31 protein performed as an importer and possessed two TMDs marked as TMD1 and TMD2, similarly to NBDs grouped into NBD1 and NBD2 in the three-dimensional structure presented in Figure 3A. In ABCT31, protein is fused between TMDs and NBDs. The ABC transporter's fundamental unit comprises all of these domains. The ABC signature motifs, Walker A (P-loop) and Walker B, which are prevalent but not limited to ABC transporters, are used to classify the NBD.

The assemblage, dynamics, surface characteristics, and thermodynamics of inorganic, polymeric, and biological systems have been studied using molecular modeling approaches. The target protein 3D framework has been developed using the SWISS-MODEL software and template-based homology modeling. The findings suggest that the ABCT31 protein seems to have a homological similarity with the 6pza 1 (ATP-binding cassette, sub-family C member 8). The highest template for homology-based modeling for ABCT31 was reported as having a sequence identical score of 33.17 percent.

The QMEAN-Z score was found to be 3.57 in the context of the global score by QMEAN, indicating the relatively strong quality of the model (<4) (Figure 3B). The model quality determinant is the Z score value representing the structure's total energy. The observed Z score value of ABCT31 was noted as −10.34, as shown in Figure 3D, which falls in the range normally found in comparable protein chains in the PDB, demonstrating the structure's dependability. The energy plot presented in Figure 3E depicts the quality of the local model by graphing energies as a function of the position of amino acids in sequence, with +ve values corresponding to problematic or incorrect regions of a model in general. The residues further validated the projected model's consistency with negative energy (Kulkarni and Devarumath, 2014).

WHAT IF was used to assess the packing environment for residues of the modeled ABCT31 to the experimental structures. An equal or < -5.0 score indicates poor structural packing (Singh et al., 2016; Stitou et al., 2021). Compared with the template X-ray structure (Figure 3C), the produced structure has similar packing scores, with only a few residues having poor packing, as their score values have been shown (−1.074) lesser than −5.0. The ABCT31's stereochemical and energetic features were evaluated using a Ramachandran plot, which revealed that 1.3 percent of residues (15) in the disallowed/outlier region, 5.6 percent of residues (66) in the allowed region, and 93.1 percent of residues (1094) in the most favored zones, of the RAMPAGE server (Figure 3E). The statistics in the preferred and allowed regions and the low proportion in the outlier show the acceptability of the *abct*31's Ramachandran plot. The PROCHECK goodness factor (G-factor) revealed important information about the distances of covalent and overall bond angles. However, after the analysis of the G-factor for the modeled ABCT31 was > 0.502, indicating that the quality of the projected model was extremely high. The G-factor and the overall Ramachandran plot properties ensured the ABCT31structure's quality (Table 3). The overall quality factor of ABCT31 was 88.1651, according to an ERRAT analysis. The quality of the 3D protein model generated in this work is confirmed by the findings acquired from multiple quality evaluation servers.

**Table 3:** PROCHECK projected statistics of the proposed three-dimensional model.

**Parameter**	**Score**
Dihedrals	−0.26
Covalent	−0.04
Overall	−0.15

### Identification of Structural Features of ABCT31

**Figure 4:**
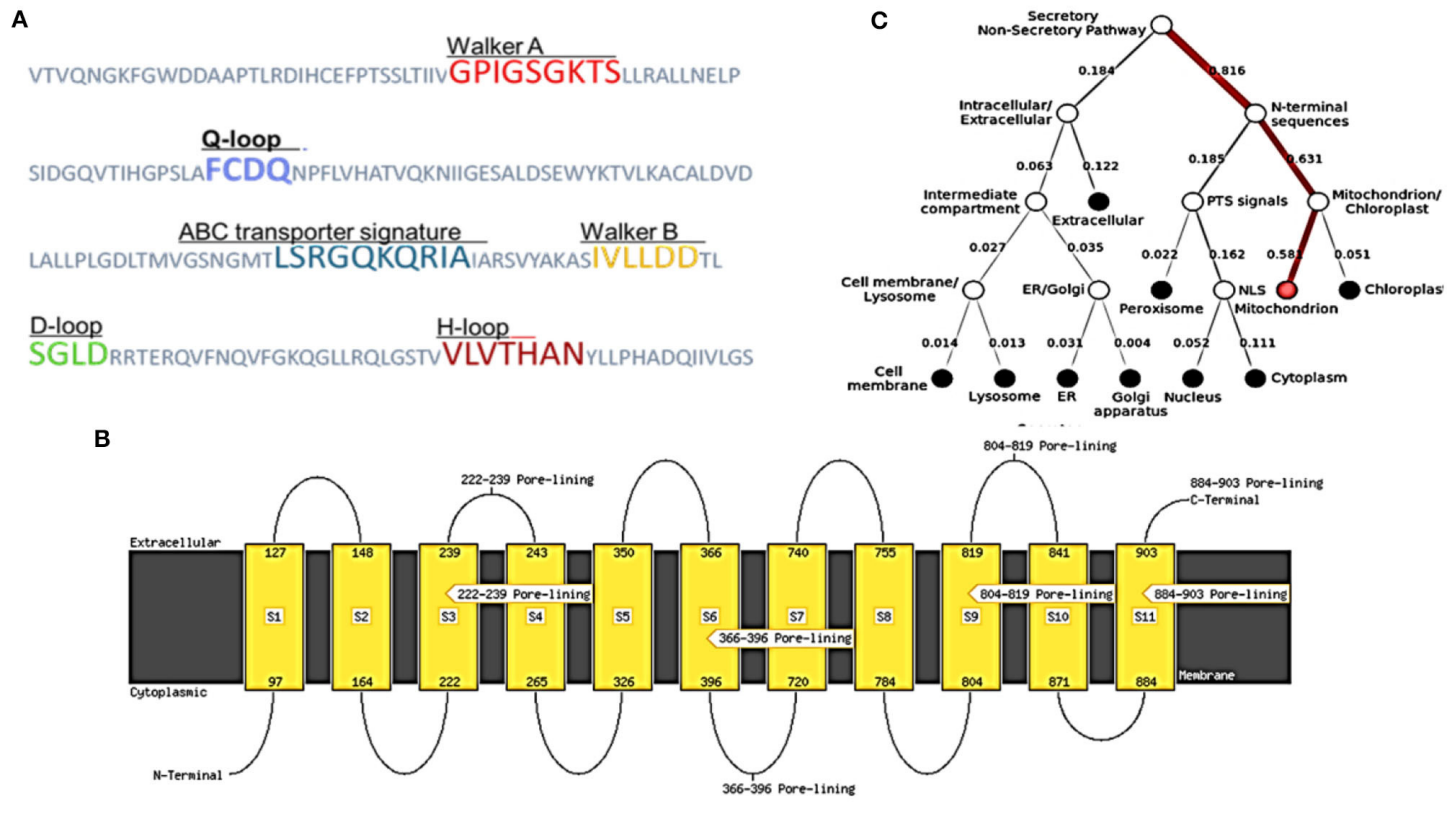
Structural features and location analysis of the ABCT31 protein. **(A)** Features loop and walker of the *abct*31 protein; **(B)** Topology analysis of the ABCT31 protein; **(C)** Location of *abct*31 gene in *M. ruber* M7.

As described in section Model Validation and Homology Modeling, the NBDs possess some conserved motifs, which play an important role in the hydrolysis and binding of ATP in the opening and closing mechanism of the TMDs'. NBDs dominate the subunits of ABC, and these might be divided into two constituent domains: the conserved Walker A motif or P-loop (GXXGXGK(S/T), which is contained in the catalytic core domain, Walker B motif (ϕϕϕϕD, ϕ represents the hydrophobic residue), switch region (also known as Q-loop and H-motif) together with an α-helical domain that is more diverse on a structural basis, and it contains the signature motif of ABC (LSXGQXXXXX). The nucleotide state affects the comparative alignment of the catalytic domains and helix. The ABC subunits were packed together in a “head-to-tail” configuration in an intact transporter so that one subunit's P loop is positioned toward the other subunit's signature. In the present ABC protein, the sequence of Walker A (GPIGSGKTS), Walker B (IVLLDD) ABC transporter signature (LSRGQKQRIA), and the three-loop structures Q (FCDQ), D (SGLD), and H (VLVTHAN) was also identified and located, respectively, as shown in Figure 4A.

Low E values (Figure 1) determined by the CDD software indicated that most amino acids in the ABC-MRP domains (accession: cd03250 and cd03244) are substantially retained in all homologs. The extremely preserved amino acid motifs are contained in the NBD of the ABC-MRP domain and play a role in generating the required energy, which is ultimately used for powering the transport mechanism (Ghilarov et al., 2021). The Walker A [GXXGXGK(S/T)] and Walker B (hhhhDE, where h represents the hydrophobic amino acid) motifs were discovered to be ATP-binding motifs inside the NBD (Figure 4A). The establishment of formally extensive connections with an ATP molecule's phosphate group is done by the Walker A motif. Similarly, the coordination of water and Mg^2+^ at the catalytic site is done by the Asp of the Walker B motif.

In contrast, the catalytic glutamate is essential for the hydrolysis of ATP (Zaitseva et al., 2005). Likewise, a conserved Gln plays an important function in coordinating the ATP molecule. This is located between the Walker sequences in the Q-loop. The identification of the conserved D-loop and switch or H-loop as additional motifs was done downstream of the Walker B motif and, respectively, termed as Asp and His. These coordinate the γ-phosphate through the collaboration of either D-loop (water molecule) or H-loop (direct hydrogen binding) (George and Jones, 2012). The Walker sequences of each NBD bearing the conserved amino acid sequence LSGGQ were also identified. This discovered subdomain is also implicated in ATP binding and is termed a helical subdomain or an ABC signature motif (Zaitseva et al., 2005; Chen et al., 2017b).

### Hydropathy Profile of ABCT31 Protein

MEMSAT SVM is a support vector machine (SVM) oriented predictor, which is very dependable software that can also predict pore-lining residues. MEMSAT 3, HMMTOP, and MEMSAT SVM are transmembrane protein topological predicting tools. With a total entropy of 18.8083, these programs indicated 11 transmembrane helices (Figure 4B). The illustrated transmembrane helices have a high number of hydrophobic amino acids, and the MEMSAT SVM software was used to represent the final interpreted topology of this transmembrane protein, as shown in Figure 4B. It also discovered four pore-lining helices with a pore stoichiometry of 1, containing residues from 222 to 239, 366 to 396, 804 to 819, and 884 to 903. It has been observed that the transmembrane domain area of transporter proteins forming transport channels is mostly made up of membrane-spanning alpha-helices with bends and kinks, giving significant structural variation that is necessary for the channel's activity (Moussatova et al., 2008; Nugent and Jones, 2012). The occurrence of transmembrane with hydrophobic regions revealed its membrane-embedded architecture and participation in transmembrane transportation, which can be confirmed further through functional and structural analysis.

### Subcellular Localization of *abct*31 Gene in *M. ruber* M7

FFPred predicted the biological function as transmembrane transport with 0.907 probability based on gene ontology. Molecular function analysis predicted transmembrane transporter activity as 0.963 and substrate-specific activity of transmembrane transporter as 0.769. However, the cellular component prediction was predicted as an intrinsic or integral part of the membrane with a probability of 1.0. Deeploc server identified that *abct*31 protein is a membrane type with 0.9973 scores while the soluble scoring is 0.0027. It also predicted that it is located in the cell membrane with a score of 0.378 (Figure 4C).

### Substrate Binding Properties of ABCT31 Protein

**Figure 5:**
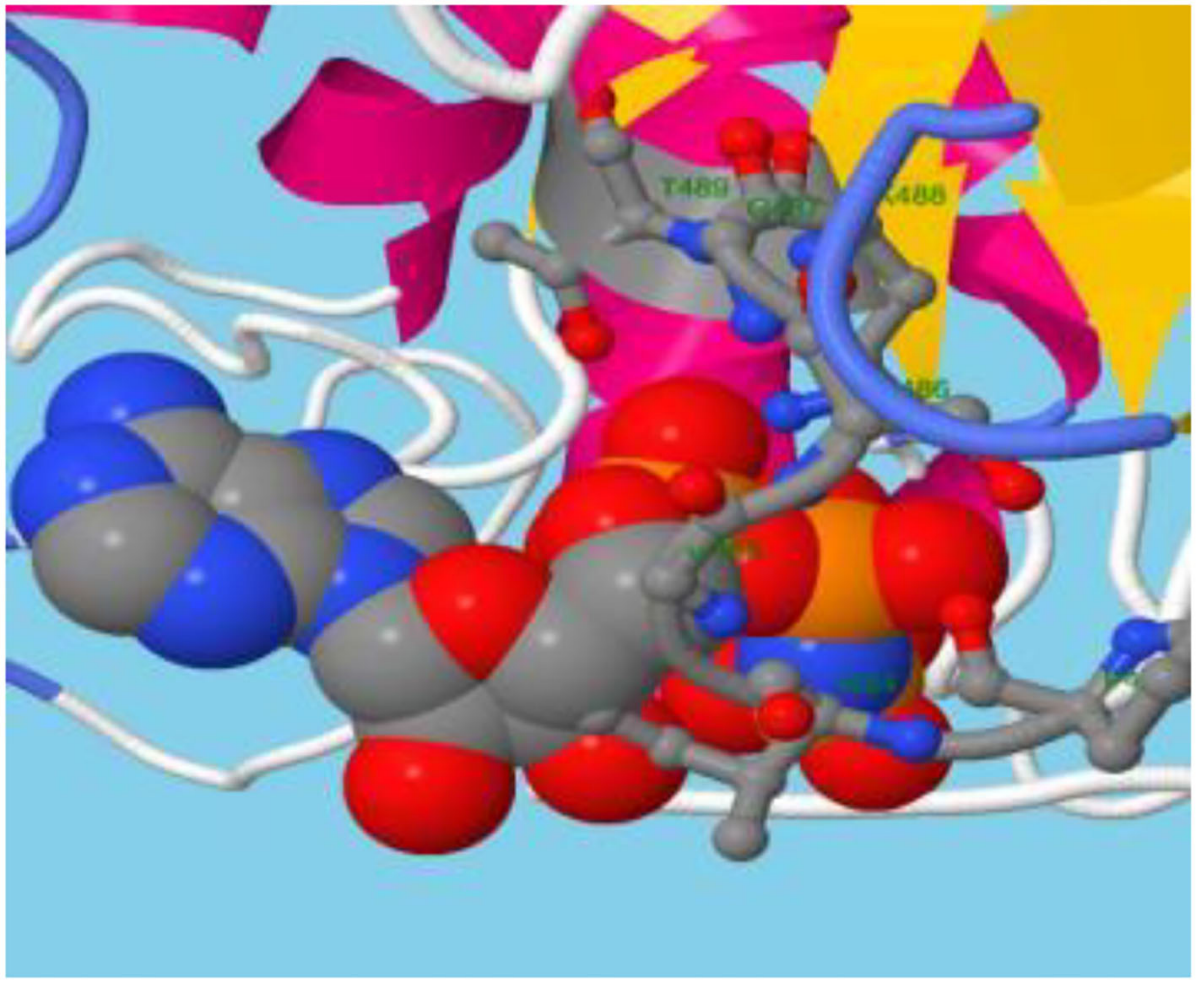
ABC transporter carries the substrate.

Structural information, outcomes of GO prediction, and domain analysis altogether recommend the localization and function of ABCT31 protein membrane in the transportation of substrates through transmembrane, especially the transportation of phenylacetic acid is needed to be studied further through the 3D structure analysis. However, another trusted online method known as the RaptorX-binding site prediction tool identified PA as the potential ligand and discovered two binding sites regulated by residues G487 P483, I484, G485, S486, K488, and T489 (Figure 5).

### Genetic Deletion of *abct*31 Gene for the Construction of Δ*abct*31

**Figure 6:**
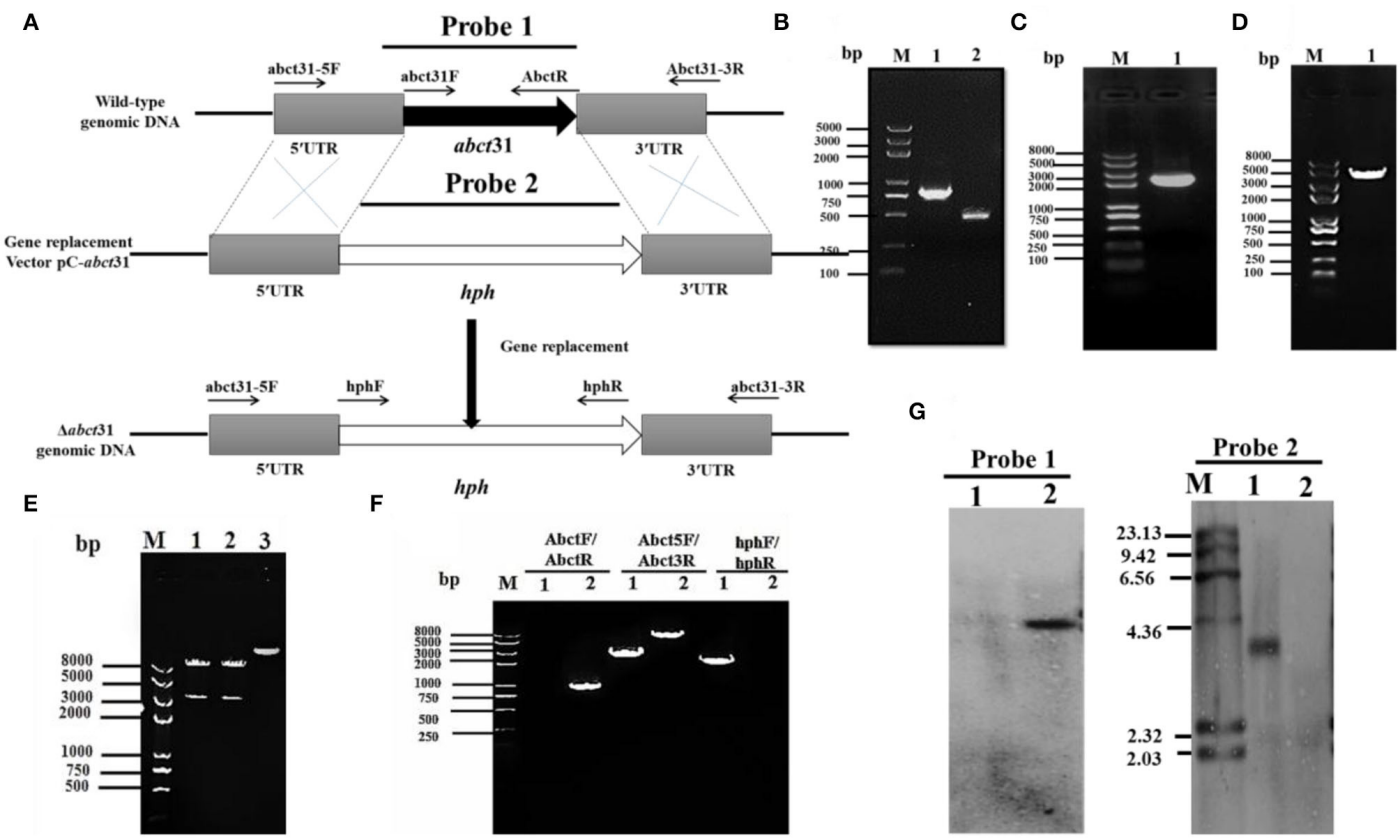
Construction and verification of Δ*abct*31 strain. **(A)** Pictorial presentation for the homologous recombination approach to create *abct*31 disruption strains. **(B)** M: Trans 2k II Marker; Lane1: PCR product of 5' franking region (770 bp); Lane 2: PCR product of 3' franking (434 bp). **(C)** M: Trans 2k plus II Marker; Lane 1: *hph* (2,137 bp). **(D)** M: Trans 2k plus II Marker; Lane 1: PCR product of recombinant fragment of 5'UTR, *hph* and 3'UTR (3341 bp). **(E)** M: Trans 2k plus II Marker; Lane1: Restriction enzyme digestion analysis of vector pC-*abct*31 (*KpnI*/*HindIII*). **(F)** Validation of *abct*31 homologous recombination events. M: Trans 2k plus II Marker; Lane 1, the *abct*31 strain; Lane 2, the wild-type strain (M7). Different distinct bands were obtained by PCR amplification for selected pair of primer. **(G)** Southern hybridization analysis. Lane 1, Xba1 digested genomic DNA of Δ*abct*31; lane 2, Xba1 digested genomic DNA of M7 respectively; M: λDNA/*HindIII* marker; Probe 1: *abct*31 gene; Probe 2: *hph* gene.

To examine the role of *abct*31 *in vivo*, the development of the *abct*31 gene disruption strain (Δ*abct*31) was done by using a method (Shao et al., 2009; Liu et al., 2014, 2019). The amplification of the upstream 5' flanking region and downstream 3' flanking region (Figure 6B) was done from the genomic DNA of *M. ruber* M7 (schematic presentation in Figure 6A). At the same time, the pKSH vector was used to amplify the *hph* gene fragment (Figure 6C). All amplified fragments were purified by gel electrophoresis and a gel purification kit (Figure 6D). During the next step, the disruption cassette was ligated with the linearized pCAMBIA3300 vector by T4 ligase to make the knock-out vector (pC- *abct*31) of *abct*31. The successful ligation was further confirmed by double digestion of pC-*abct*31 (Lane 1-2); two bands, 8.0 kb by pCAMBIA3300 and 3.3 kb of disruption cassette, are presented in Figure 6E.

The six putative deleted (Δ*abct*31) strains obtained by *Agrobacterium*-mediated T-DNA transformation to *M*. *ruber* M7 as a recipient were also identified and authenticated by PCR analyses. One of the mutants was subjected to further PCR verification, and the results are as shown in Figure 6F. The 3 pairs of primers Abct31*-*5F/Abct31*-*3R, Abct31F/Abct31R, and hphF/hphR (Table 1) used for the validation of the homologous recombination event. Lane 2 for the M7 genomic DNA and Lane 1 for the Δ*abct*31 strain, the primer pair Abct5F/Abct3R 3.3 kb and 6.0 kb band, were amplified, respectively. In the case of ORF prime pair Abct31F/Abct31R, nothing was amplified from the Δ*abct*31 strain while the 800 bp band amplified M7 genomic DNA. Controversially, the hphF/hphR primer pair 2.1 kb band appeared in Lane 1 (Δ*abct*31 strain), and nothing was seen in Lane 2. The above PCR results are demonstrated in Figure 6F, the main differences in genomic DNA of Δ*abct*31 strain and *Monascus ruber* (wild type) strain.

Furthermore, performing the transformant Southern hybridization verification, the results are shown in Figure 6G; with Probe 2 (Table 1) as a probe, a single hybridization band appeared in the transformant Δ*abct*31 and nothing appeared in M7 DNA. However, ORF (Table 1) was used as a Probe 1; no hybridization band appeared for Δ*abct*31 strain. Hybridization bands indicate that the *abct*31 gene in the mutant has been successfully replaced by the single copy of the *hph* resistance gene.

Many genes involved in the manufacture of pigments, antibiotics, herbicides, and other secondary metabolites have been cloned in the previous decade. The biosynthetic genes for cephalosporin, penicillin, cephamycins, and penicillin are grouped in clusters, as are the biosynthetic genes for other secondary metabolites and antibiotics (Ullán et al., 2002). Homologous recombination (HR) is the core tool in DNA repair that is efficiently used for gene knockout from any part of DNA (Aguilera and Gómez-González, 2008). This study shows that HR is an extremely useful tool for gene removal in filamentous fungi. The *abct*31 encodes the ABC transporter in the β-Lactam gene cluster of M7. The Δ*abct*31 (deletion mutant) of this gene is created by using the gene disruption cassette (5'UTR-*hph*-3'UTR). In this cassette, the *hph* is used as a resistant gene for the screening of the mutant. The cassette's 5'UTR (770 bp) and 3'UTR (434 bp) fragments are amplified from the upstream and downstream regions of the gene, respectively. Further verification of the gene's role in *abct*31 β-lactam gene cluster of M7 has been conducted by evaluating the relative expression level toward weak organic acid.

### Phenotypic Characterization of Δ*abct*31

**Figure 7:**
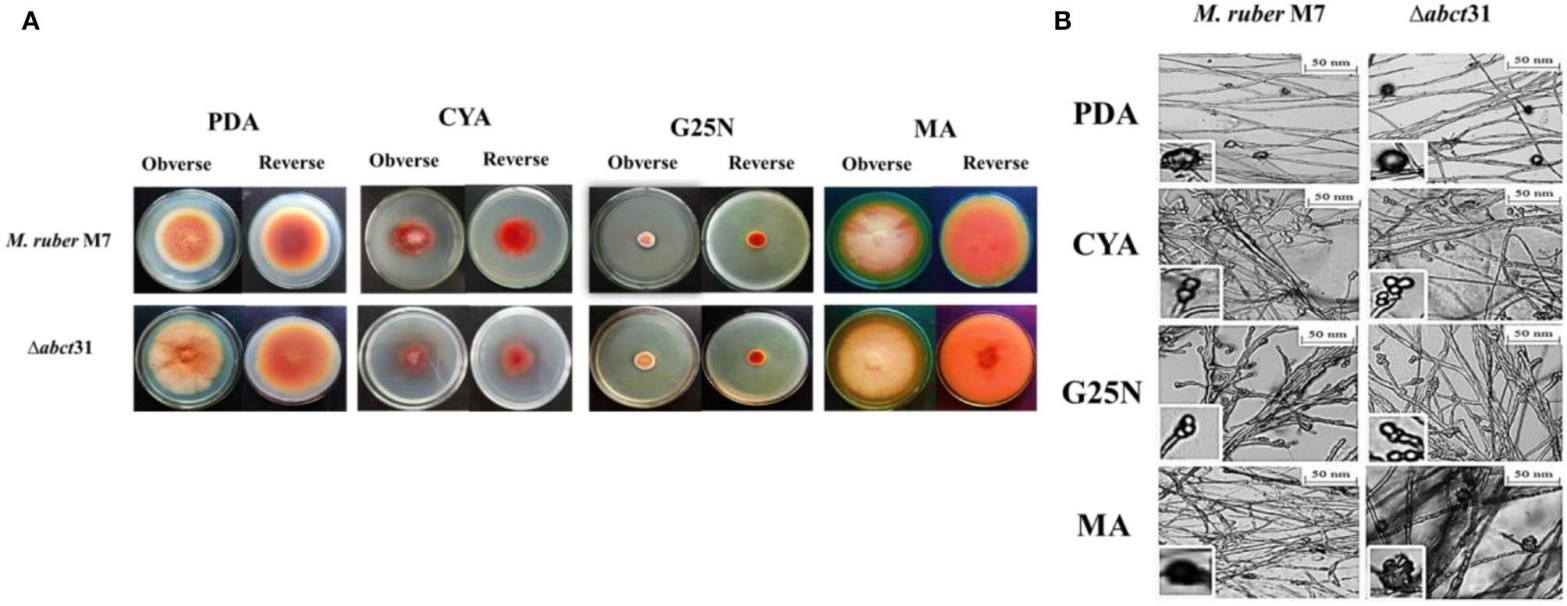
Morphological comparison of *M. ruber* M7and Δ*abct*31. **(A)** Colony morphology of *M. ruber* M7 and Δ*abct*31 on PDA, CYA, G25N, MA plates, and cultured at 28°C for 15 days; **(B)** Cleistothecia and conidia development in *M. ruber* M7and Δ*abct*31 inoculated on PDA, CYA, G25N, MA plates, and cultured at 28°C for 8 days.

The phenotypical structures were observed to explore the morphological development differences between the Δ*abct31* strain as related to *M. ruber* M7. Both deletion and wild-type strains were inoculated on four different media, including PDA, G25N, CYA, and MA, and cultivated at 28°C for 15 days. The colony size, color, and other morphological properties observed (Figure 7A) are commonly applied to investigate the morphological characteristics of *Monascus* strains (Li et al., 2010, 2014).

While seeing the results of *M. ruber* M7 and *abct*31 deletion strain (Δ*abct*31), it was observed that the colonies of Δ*abct*31 normally grew when these were compared with colonies of wild-type *M. ruber* M7 for all media (Figure 7A). However, there was a substantial difference between the strains on PDA and MA medium plates in terms of phenotypic analyses, such as colony appearance, colony edges, the size of PDA, colony diameter, and growth rate (Figure 7A). But when it comes to CYA, Δ*abct*31's colony color was darker than *M. ruber* M7's. There were slight changes in the colony color and size of Δ*abct*31 when compared with *M. ruber* M7 in G25N.

From Figure 7B, it can be seen that the Δ*abct*31 mutant can produce a cleistothecium compared with *M. ruber* M7 on PDA and MA. There was no change observed in the cleistothecia growth pattern and size. However, the conidia structure can be observed in CYA and G25N. In both media, the conidia were observed in normal shape and size. It was clearly noted that no difference was found in the quality and quantity of cleistothecium and conidium (Figure 7B). As a result, there were no differences in cleistothecia and conidia's overall development and phenotypic characteristics between the Δ*abct*31 (mutant strain) and *M. ruber* M7 (wild strain).

As well as considering the growth and structure of the hyphae in mutant Δ*abct*31 strain remained unchanged compared with the control of *M. ruber* M7. There were no structural abnormalities found in mycelia regarding the diameter and shape. Moreover, the branching pattern and growth of the mycelia also typically look normal in spreading form, as shown in Figure 7B.

#### Phenotypic Characterization of Δ*abct*31 for Feeding Precursor Amino Acids

**Figure 8:**
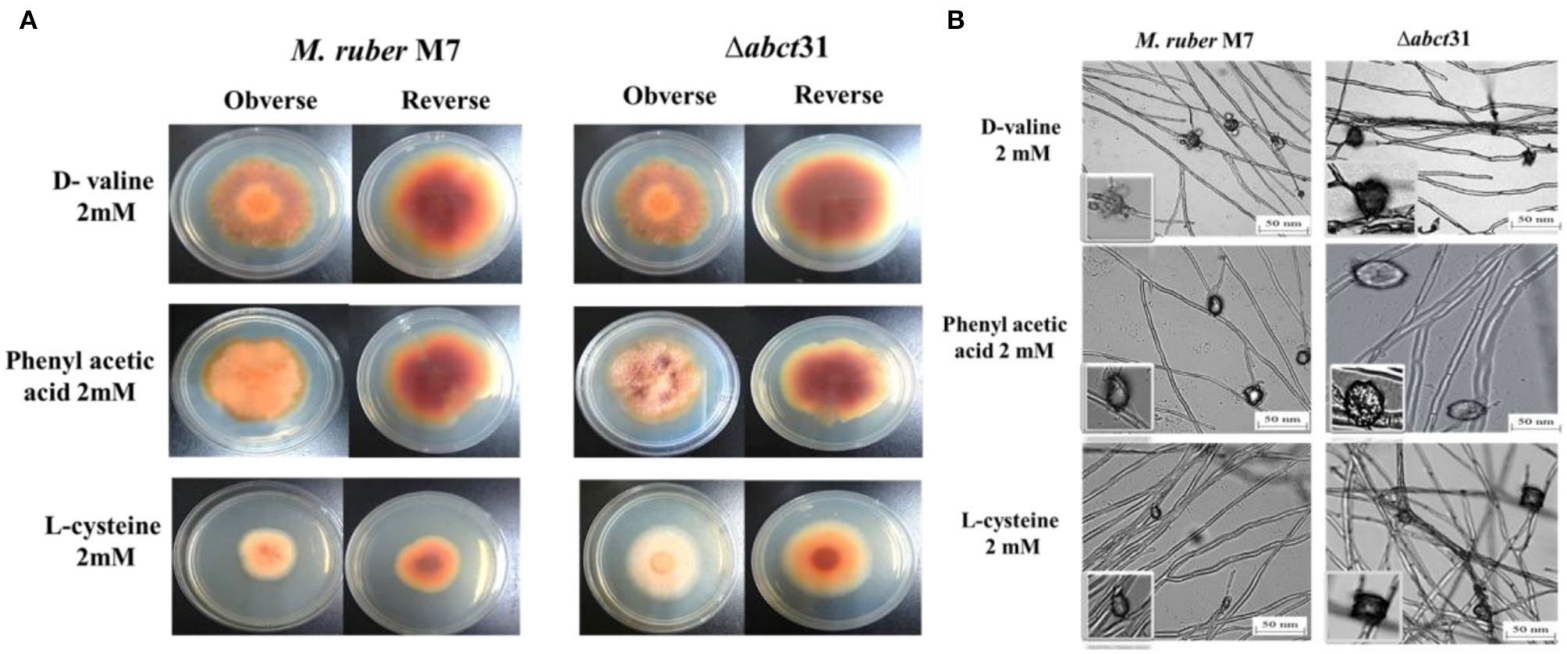
Morphological comparison of *M. ruber* M7and Δ*abct*31 to evaluate the sensitivity toward pathway amino acid supplementation. **(A)** Colony morphology of *M. ruber* M7 and Δ*abct*31 inoculated on PDA plates supplemented with D-valine, phenylacetic acid, and L-cysteine at 2 mM concentrations for each cultured at 28°C for 15 days. **(B)** Cleistothecia and conidia development in *M. ruber* M7 and Δ*abct*31 cultured on PDA plates which supplemented with D-valine, phenylacetic acid, and L-cysteine at 2 mM concentrations for each and cultured at 28°C for 10 days.

Initially, the morphological changes in the development of the colony and pigments of the *M. ruber* M7 and Δ*abct*31 strains were explored against PAA, L-cysteine, and D-valine at 2 mM concentration (Figure 8A). The effect on the development of conidia and cleistothecia was also perceived (Figure 8B).

Whereas seeing the outcomes of *M. ruber* M7 and Δ*abct*31, a substantial difference was detected related to the phenotypic characteristics, such as the size and appearance of the colony and the colony growth rate and diameter. Considering the Δ*abct*31 on PDA supplemented with PAA, the colony size exhibits resistance to growth, the colony color is clearly reddish, and colony edges are dissimilar from the *M. ruber* M7 on the PDA-PAA plate. Similarly, from colony diameter, the Δ*abct*31 displayed less sensitivity toward L-Cys supplementation. Moreover, colony color is a pale orange on the PDA-L-Cys feed plate for Δ*abct*31 when compared with wild-type *M. ruber* M7 and colony diameter is higher than wild type (Figure 8A). On the PDA-D-Val plate, the Δ*abct*31 mutant colony color and appearance look similar to *M. ruber* M7 (Figure 8A). Hence, Δ*abct*31 has a strong sensitivity toward PAA feeding.

From the results shown in Figure 8B, it can be seen that the Δ*abct*31 can produce a cleistothecium compared with *M. ruber* M7 on PDA for the supplementation of all pathway precursors. It was clearly noted that there is no difference in the quantity of cleistothecium for all precursors. Furthermore, when comparing the mutant strains to the wild strain for D-Val and L-Cys, there were no differences in general development and phenotypic of cleistothecia. However, in the case of PAA feeding, the cleistothecia size increased compared to the wild type. Besides, considering the growth and structure of the hyphae in mutants Δ*abct*31 strains looks normal and parallel to the control of *M. ruber* M7 for all precursors. No structural defects such as weakening or inflammation were found in the hyphae of the mycelium by supplementation of precursors.

Moreover, the branching pattern and growth of the mycelia were also typically scattered, as presented in Figure 8B. Hence, the feeding of pathway precursors such as D-Val, and L-Cys at 2 mM has no effect on the Δ*abct*31 regarding microscopic structures. In contrast, Δ*abct*31 exhibits a sensitivity toward PAA feeding regarding cleistothecia size.

#### Effect of *abct*31 Gene Deletion on Biomass Production Without and With the Feeding of Precursor Amino Acids

**Figure 9:**
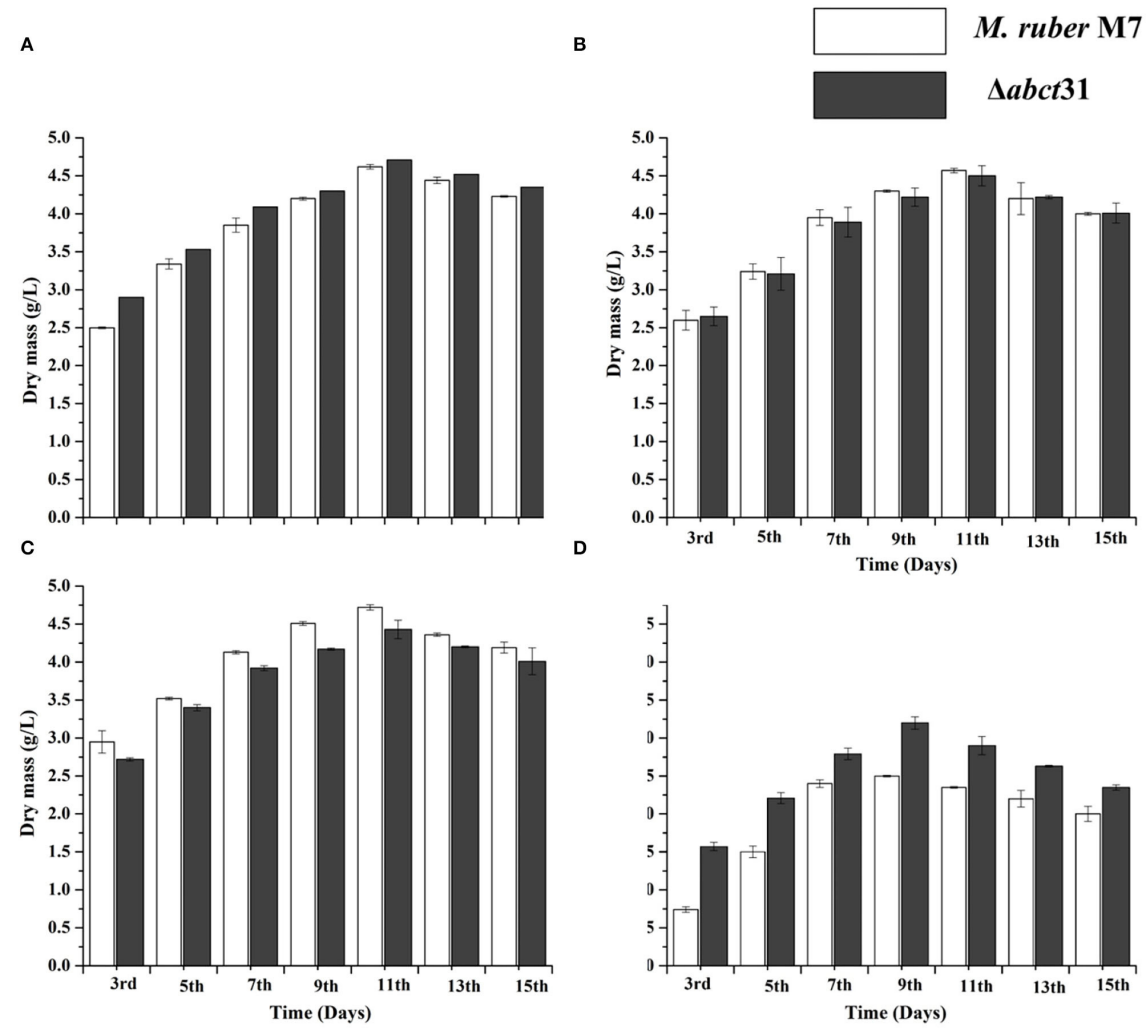
Comparison of biomass (dry cell weight) of the *M. ruber* M7 and Δ*abct*31 against pathway amino acid. **(A)** PDB medium without amino acid (control); **(B)** PDB medium with D-valine; **(C)** PDB medium with phenylacetic acid; **(D)** PDB medium with L-cysteine, at 2 mM concentrations and incubated at 28°C without agitation. The bar representing the mean of triplicate values and error bars show standard deviation.

The biomasses (dry mycelium) of the *M. ruber* M7 and Δ*abct*31 were appraised employing the gravimetric process. All strains were uniformly spread on PDA plates covered with cellophane-sheet without and with supplementation of the phenylacetic acid, L-cysteine, and D-valine at 2 mM concentration, cultivated at 28°C. The mycelia were collected at a specific time interval and then dried in an oven at 60°C until constant weights were obtained. The average weight of biomass was calculated and presented in Figure 9.

The samples of biomass of *M. ruber* M7 and Δ*abct*31 cultured on PDA and incubated at 28°C for 15 days were collected on alternate days for the 3rd−15th day for biomass comparison. The mean of the triplicate data is represented by the bar, while the error bars illustrate the standard deviation. By weighing the weight of the dried mycelia, the biomass (dry mass) of *M. ruber* M7 (control) to Δ*abct*31 strain was determined. The data shown in Figure 9A revealed a substantial (*p* < 0.05) increase in biomass values for both strains up to the 15th day without supplementation. However, the highest dry cell weight was found on the 11th day, and onwards, the inclination in biomass was observed.

The biomass results presented in Figures 9B–D reveal that among the precursors such as D-Val, PAA, and L-Cys feeding fermentation, a variation in biomass production was observed for mutant Δ*abct*31 when compared with the wild type (*M. ruber* M7). However, mycelium production starts to increase after the 3rd day of fermentation. The results are presented in Figure 9B; for the D-Val, feeding fermentation showed a non-significant reduction in the biomass for Δ*abct*31 when compared with the *M. ruber* M7, up to 11th d; for PAA feeding (Figure 9C), the biomass for Δ*abct*31 was significantly lower when compared with the *M. ruber* M7 until the 15th d, and significantly (*p* < 0.01) higher biomass was observed in the L-Cys feeding case for Δ*abct*31 when compared with *M. ruber* M7 (Figures 5–9D). However, the results presented in Figures 9B,C exhibited a significant (*p* < 0.05) increase in the biomass quantity for Δ*abct*31 and *M. ruber* M7 up to 11th d for D-Val and PAA, at 9th d of L-Cys supplementation, shown in Figure 9D, and a clear reduction in the biomass value was shown in L-Cys < PAA < D-Val precursors. Overall, in the L-Cys feeding experiment, biomass production value was the lowest compared to other precursors.

#### Effect of *abct*31 Gene Deletion on the Production of Pigments Without and With the Feeding of Precursor Amino Acids

*Monascus* spp. can yield numerous secondary metabolites (Chen et al., 2017b), especially *Monascus* pigments (MPs) such as red (Monascorubramine and Rubropunctamine), orange (Monascorubrin and Rubropunctatin), and yellow (Ankaflavin and Monascin). To check the effect of Δ*abct*31 gene deletion on MPs, the MPs profile was detected without and with supplementation of the phenylacetic acid, L-cysteine, and D-valine, at 2 mM. Table 4 presents the MPs' average results. The production of the overall pigment rose significantly (*p* < 0.01) from days 3 to 15 for all strains.

**Table 4:** Comparison of pigments production of *M. ruber* M7 and Δ*abct*31 feeding D-Val, PAA, L-Cys.

**Days**	***M. ruber* M7**	**Δ*abct*31**
3rd	42.79 ± 0.27^g^	41.92 ± 0.18^g^
5th	70.59 ± 0.13^f^	72.83 ± 0.15^f^
7th	86.36 ± 0.15^e^	89.87 ± 0.08^e^
9th	94.58 ± 0.14^d^	98.63 ± 0.11^d^
11th	103.32 ± 0.22^c^	106.24 ± 0.05^c^
13th	110.31 ± 0.17^b^	114.33 ± 0.13^b^
15th	117.04 ± 0.20^a^	120.27 ± 0.17^a^
3rd	42.79 ± 0.27^g^	41.92 ± 0.18^g^
**D-Val**		
3rd	22.63 ± 0.45^g^	26.33 ± 0.20^g^
5th	40.74 ± 0.11^f^	42.02 ± 0.10^f^
7^t^h	56.36 ± 0.32^e^	58.44 ± 0.08^e^
9th	64.58 ± 0.29^d^	66.86 ± 0.12^d^
11th	78.32 ± 0.36^c^	80.12 ± 0.09^c^
13th	84.31 ± 0.31^b^	87.86 ± 0.08^b^
15th	95.04 ± 0.32^a^	98.94 ± 0.28^a^
**PAA**		
3rd	52.80 ± 0.05^g^	52.23 ± 0.03^g^
5th	87.11 ± 0.06^f^	92.54 ± 0.10^f^
7th	106.56 ± 0.07^e^	110.95 ± 0.04^e^
9th	116.71 ± 0.10^d^	119.46 ± 0.09^d^
11th	121.32 ± 0.23^c^	125.67 ± 0.06^c^
13th	138.72 ± 0.04^b^	139.93 ± 0.03^b^
15^th^	144.43 ± 0.03^a^	146.90 ± 0.08^a^
**L-Cys**		
3rd	14.75 ± 0.22^g^	19.93 ± 0.14^g^
5th	17.15 ± 0.05^f^	21.06 ± 0.11^f^
7th	23.45 ± 0.20^e^	28.12 ± 0.03^e^
9th	28.44 ± 0.14^d^	32.84 ± 0.02^d^
11th	34.49 ± 0.27^c^	39.97 ± 0.11^c^
13th	39.86 ± 0.20^b^	43.09 ± 0.09^b^
15^th^	42.81 ± 0.27^a^	47.13 ± 0.05^a^

*Analysis for comparing the pigment production in M. ruber M7 and Δabct31 mutant cultivated on PDA without and with supplementation of the phenylacetic acid, D-valine, and L-cysteine at 2 mM concentration, incubated at 28°C up to 15 d, mycelia and medium were collected from 3rd to 15th d with an alternate day interval. The values are represented as the mean of extracellular and intracellular values ± sd*.

When any strain is grown in a liquid media, it produces metabolites and transports them to growth media via a well-defined transport system. After the fermentation of Δ *abct*31 and wild-type strains in PDB, both extracellular and intracellular contents of pigments were observed. The overall means of extracellular and intracellular MPs were calculated. The results (Table 4) showed that MPs slightly increased in Δ*abct*31 for control and D-Val, PAA, and L-Cys precursor feeding, during PDB fermentation duration from 3rd to 15th d. The slight increase of MPs of Δ*abct*31 might be due to their role in transporting intermediates. This may be due to the side chain amino acid level increased in the medium; more substrates are available for the pigment production.

### Detection of PG by HPLC

The HPLC was used to study the involvement of the *abct*31 gene for penicillin production in *M. ruber* M7 and deletion (Δ*abct*31). Furthermore, the role of the feeding of precursors, i.e., D-Val, PAA, and L-Cys at 2 mM on PG production in both strains was also observed. The samples collected on the 7th day, in addition to the treated samples, were analyzed by HPLC for PG estimation.

**Figure 10:**
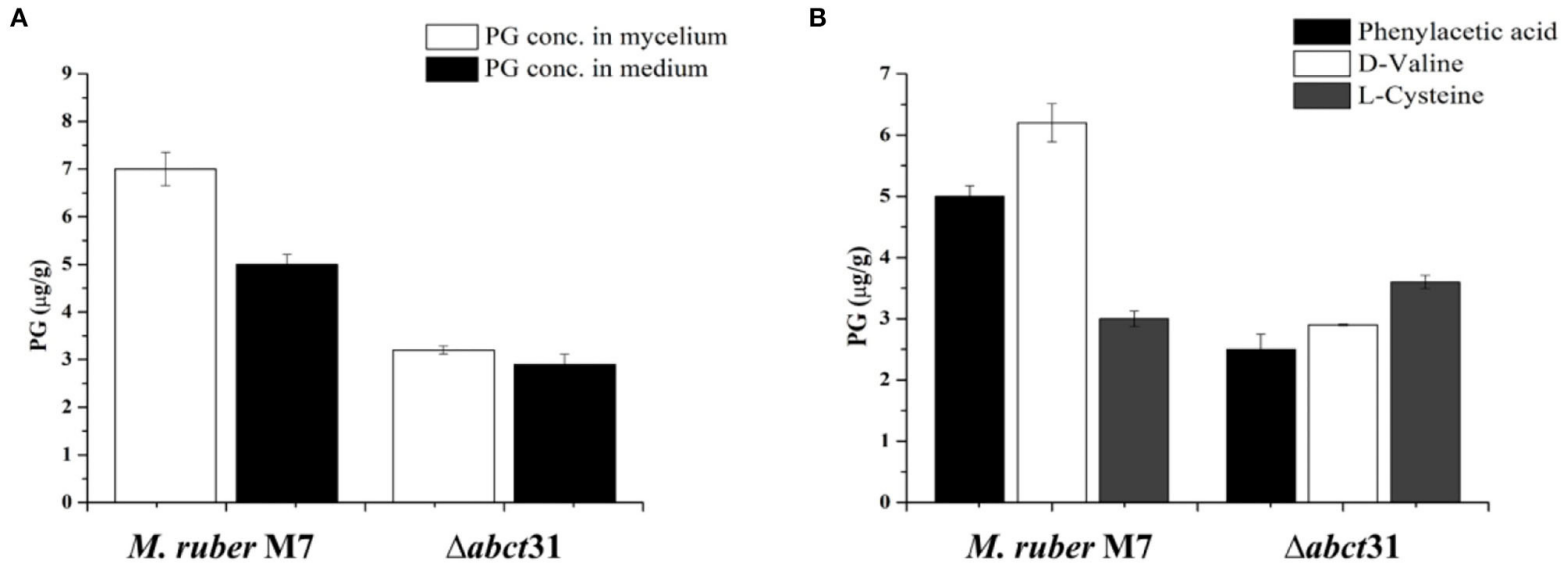
Comparison of PG concentration of the wild-type strain and the Δ*abct*31 transformants on the 7th day. **(A)** PG concentration in mycelium and medium; **(B)** Comparison of PG concentration affected by supplementation of D-valine, phenylacetic acid, and L-cysteine at 2 mM on 7th day. The bar representing the mean of triplicate values and error bars show standard deviation.

In the Δ*abct*31 strains, the PG level was observed in intracellular and extracellular. However, the HPLC results showed that these strains could produce the PG at the intracellular level and in the extracellular medium for the 7th day (Figure 10). But the concentration of PG is significantly decreased in the extra- and intracellular part of the mutant when compared with *M. ruber* M7. The average yield of PG in transformants' (Δ*abct*31) intracellular portion is calculated as 3.2 ± 0.08 μg/g, when compared with the parental strain's yield (Figure 10A). However, the extracellular results showed a similar pattern for PG production Δ*abct*31, yielding the PG, 2.9 ± 0.21 μg/g when compared with the parental strain (Figure 10A).

From Figure 10B, it has been observed that the individual feeding of D-Val, PAA, and L-Cys at 2 mM concentration has a substantial impact on the *M. ruber* M7. The highest PG concentration was 6.20 ± 0.17 μg/g observed for the D-Val feeding, in contrast to the L-Cys the lowest PG level (3.23 ± 0.12 μg/g). However, for Δ*abct*31 mutants, the lowest PG production of 2.50 ± 0.24 μg/g in Δ*abct*31 mutants has been detected for PAA feeding. While among these precursors feeding in Δ*abct*31, the highest PG level was observed for L-Cys 3.64 ± 0.10 μg/g, presented in Figure 10B. From all results of feeding experiments, it has been concluded that Δ*abct*31 has the highest sensitivity toward PAA precursor.

### Metabolic Profile by Ultra-Performance Liquid Chromatography (UPLC)

**Figure 11:**
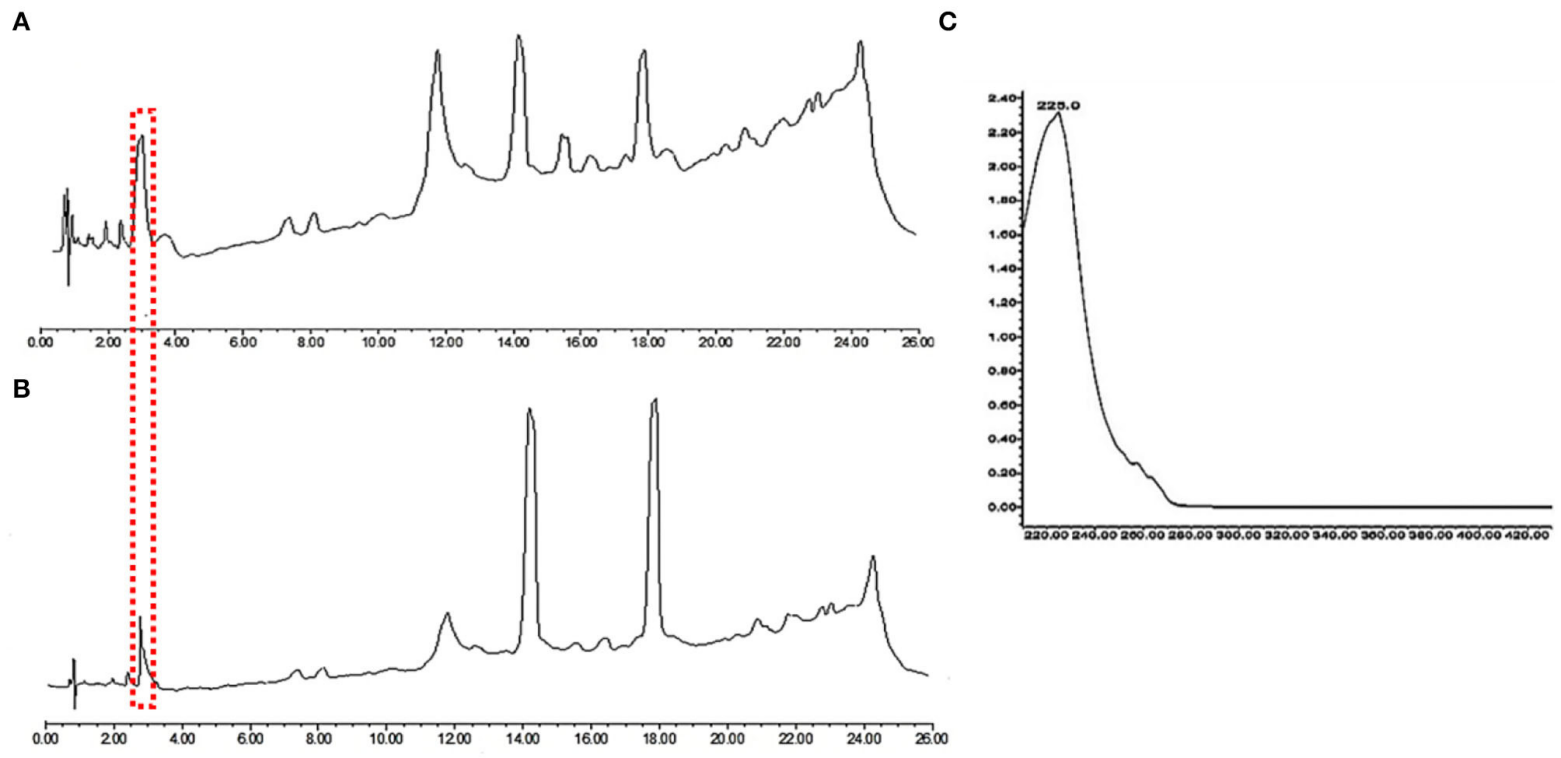
Comparison of eluted products of *M. ruber* M7 and Δ*abct*31 transformant for solid-state fermentation on rice. **(A)** Metabolic profile of *M. ruber* M7 at 250 nm; **(B)** Metabolic profile of Δ*abct*31 at 250 nm; **(C)** UV-Vis spectrum of benzyl penicillin (PG) which is shown by the arrows in metabolic profiles.

In order to investigate further the difference in the metabolic profile between *M. ruber* M7 and mutant (Δ*abct*31), ultra-performance liquid chromatography (UPLC) was used for more elaboration of the HPLC results. The results are shown in Figure 11. The penicillin G spectrum is shown in Figure 11C. As shown in Figures 11A,B, the peak appearance for penicillin G took place at around 3.8 min for sample filtrates of *M. ruber* M7 and Δ*abct*31, respectively. The UV-Vis spectra confirm the presence of PG in both strains; however, the overall peak area was reduced. Hence, *abct31* might be involved in the transportation of intermediate (IPN and PAA) compounds to produce PG in *M. ruber* M7. For additional validation about the function of the *abct*31 gene regarding the PG production, UPLC-MS/MS has been performed.

### Detection of β-Lactam Metabolites in Metabolic Profile Using UPLC-MS/MS

**Figure 12:**
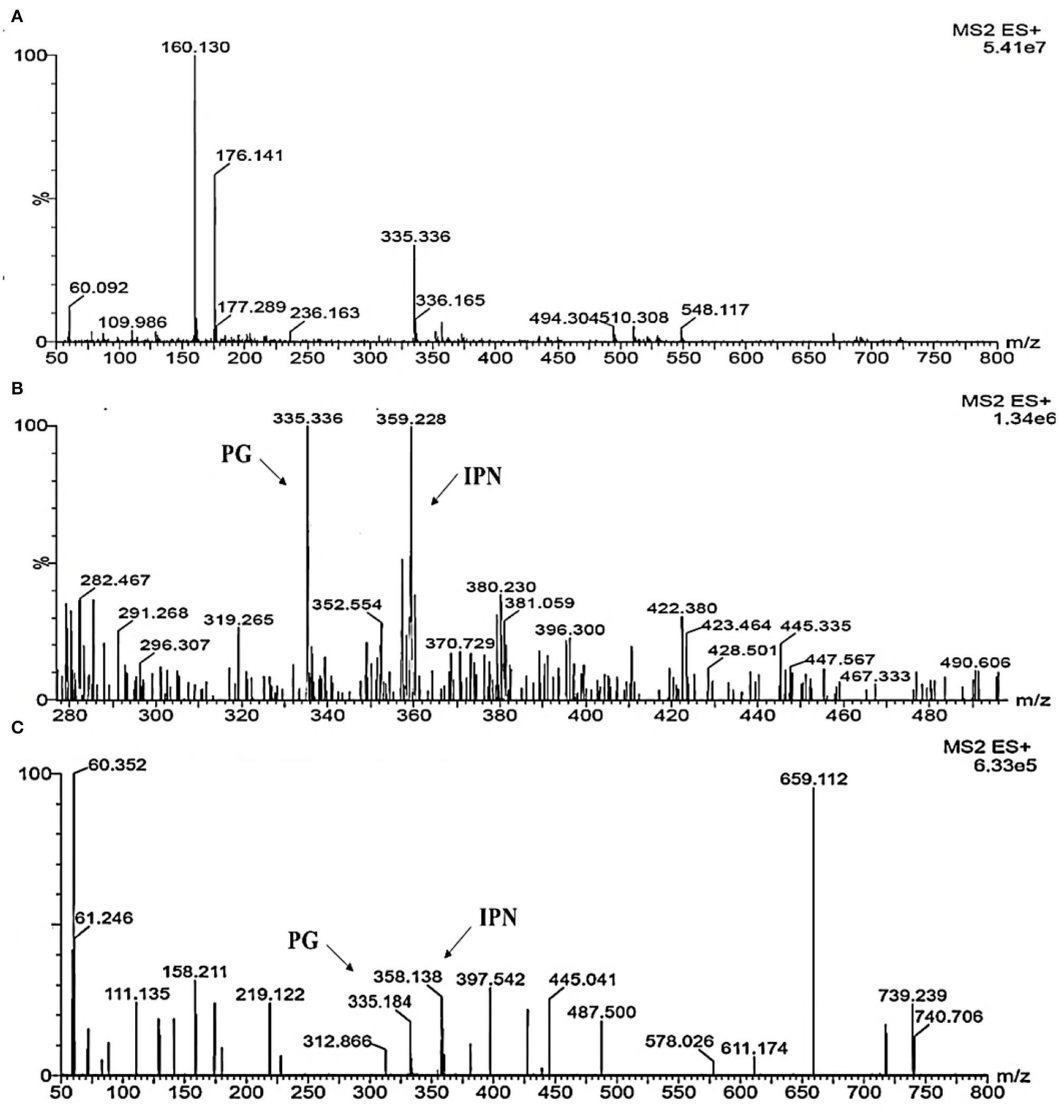
Detection of β-metabolites detected *M. ruber* M7 and Δ*abct*31 transformants by UPLC-MS/MS. UPLC-MS/MS spectrogram of the *M. ruber* M7 and Δ*abct*31 for solid-state fermented on rice displays that the atomic mass of ion (m/z 335.336) matched well with the standard penicillin, the molecular weight for isopenicillin N is 359.3 g/mol, in spectrogram ion m/z 359.00 representing the isopenicillin N peak. **(A)** Spectra in A penicillin G (PG) standard with the mass 335.336; **(B)** A pattern of fragmentation of *M. ruber* M7; **(C)** A pattern of fragmentation of Δ*abct*31.

To investigate the metabolites, a mass profile of *M. ruber* and Δ*abct*31 was developed using the rice (solid-state fermentation) filtrate. Because the filtrate revealed a wider range of biological compounds, Figure 12 shows the findings of the footprinting of the numerous metabolites found in *M. ruber* M7.

According to Figure 12, molecular masses associated with β-Lactam such as PG and their intermediates IPN were discovered in the culture filtrate. As a result, PG and IPN in the extract *M. ruber* M7 were also observed in the UPLC-MS/MS study. The existence of the 335.336 m/z peaks for PG and 395 m/z for isopenicillin N in the crude extract of M7 (Figure 12B) and alike fragmentation design present in the penicillin G standard (Figure 12A) has confirmed the identity of this metabolite. Moreover, as Figure 12C shows that the PG and IPN peaks were also detected in Δ*abct*31.

From the above results, the presence of the IPN peak in Figure 12C demonstrated that *abct*31 might be responsible for the transportation of intermediate, phenylacetic acid. For further clarification, the sensitivity toward weak acids has also been evaluated.

### Weak Acid Sensitivity

The toxicity of PAA with weak acids was tested for Δ*abct*31 and in the *M. ruber* M7 since the Δ*abct*31 gene was substantially expressed when the parental strain was cultured in the presence of PAA. Herein, the spores of *M. ruber* M7 and Δ*abct*31 were plated on PDA with the supplementation of increased concentrations of sorbic acid, acetic acid, and adipic acid at pH 6.2. Further analyses were done for the colony morphologies and relative expression.

#### Colonial Sensitivity of *abct*31 Toward Weak Acids

**Figure 13:**
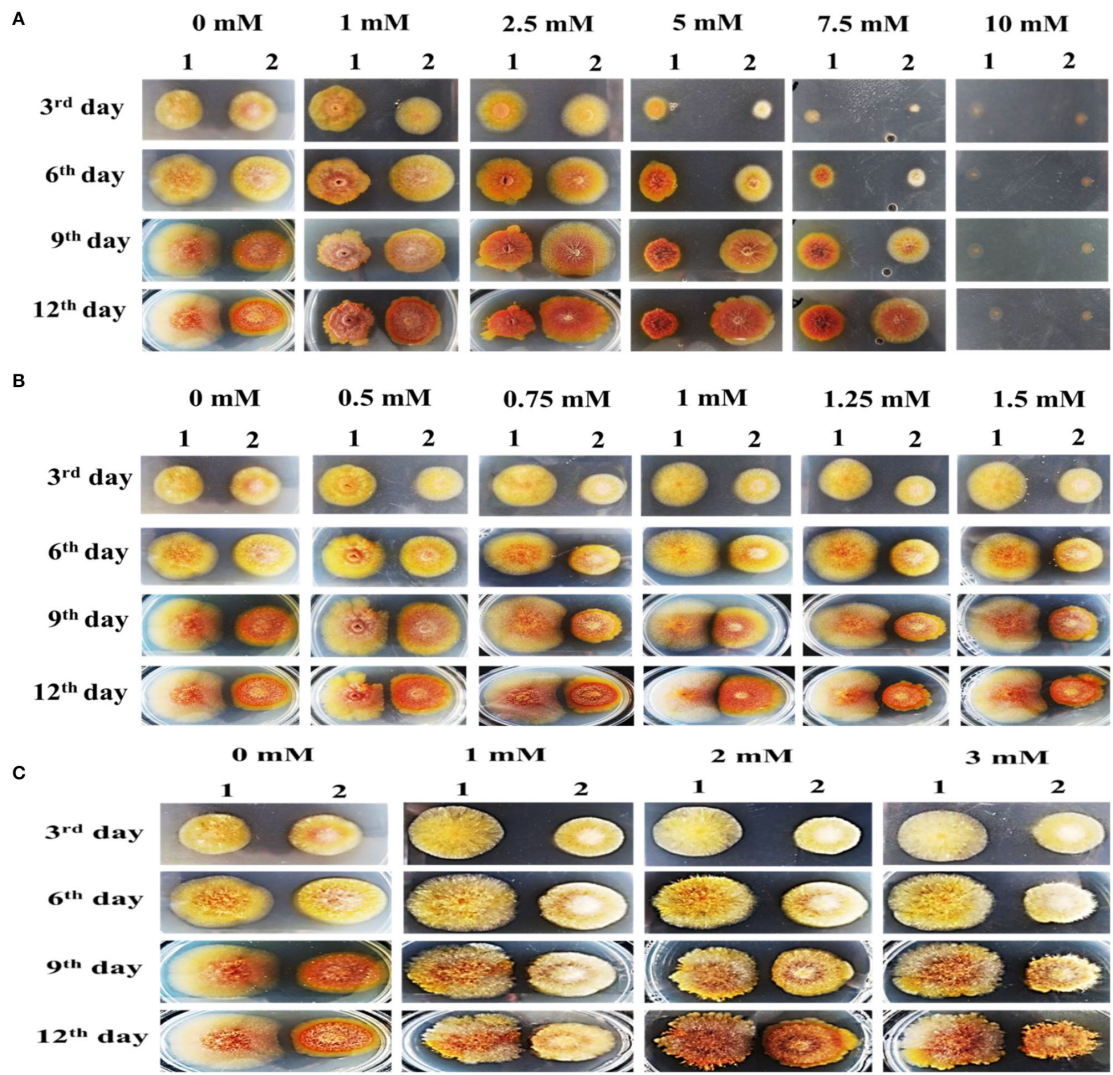
Colony morphology of *M. ruber* M7 and Δ*abct*31 for the feeding of weak acids. The morphologies of *M. ruber* M7 (1st vertical row) and Δ*abct*31 (2nd vertical row) colonies on PDA plates supplemented with weak acid at 28°C for 12d. **(A)** Colony morphology of *M. ruber* M7 and Δ*abct*31 for the feeding of sorbic acids at 0, 1, 2.5, 5, 7.5, and 10 mM concentration, respectively; **(B)** Colony morphology of *M. ruber* M7 and Δ*abct*31 for the feeding of acetic acids at 0, 0.5, 0.75, 1, 1.25, 1.5 mM concentration, respectively; **(C)** Colony morphology of *M. ruber* M7 and Δ*abct*31 for the feeding of adipic acids at 0, 1, 2, and 3 mM concentration, respectively.

To evaluate the difference in the colonies between Δ*abct*31 and *M. ruber* M7, both of them were inoculated on individual PDA plates supplemented with sorbic acid at different concentrations (0, 1, 2.5, 5, 7.5, 10 mM) with acetic acid (0, 0.5, 0.75, 1, 1.25, 1.5 mM) concentrations and also with adipic acid at different concentration levels (0, 1, 2, 3 mM) and then incubated for 12 d at 28°C; the colony sizes, color, and other morphological properties were observed (Figure 13).

The results in Figure 13A have shown that Δ*abct*31 colonial sizes were bigger than those of M7 for all tested concentrations of sorbic acid, and Δ*abct*31 colonial colors were a little bit light when compared with *M. ruber* M7 when the concentrations of sorbic acid were (1, 2.5, 5, 7.5 mM). Although the colonial sizes of *M. ruber* M7 were reduced as the concentrations of sorbic acid were increased, the colonial colors seemed reddish. The colonial edges were dissimilar from the controlled growth of *M. ruber* M7. Similarly, from colonial diameters, it was observed that Δ*abct*31 displayed less sensitivity for sorbic acid supplementation. At the highest concentration of sorbic acid 10 mM, both strains did not show any growth up to 12d (Figure 13A).

The observation in Figure 13B shows that when compared with wild-type *M. ruber* M7, the colonial sizes of Δ*abct*31 on PDA supplemented with acetic acid were smaller for higher concentrations, such as 0.75, 1, 1.25, and 1.5 mM, and the colonial color was darker than *M. ruber* M7. In the M7 strain, colonial size exhibited resistance to growth, and the colonial color was clearly lighter on the PDA-acetic acid plate at higher concentrations of 1–1.5 mM. In comparison, at a lower concentration of acetic acid (0.5 mM), the colonial size looked similar in contrast to higher concentrations. Hence, the Δ*abct*31 strain exhibited acetic acid supplementation sensitivity at higher concentrations than *M. ruber* M7 (Figure 13B).

The results shown in Figure 13C illustrate that Δ*abct*31 colonial sizes were smaller than those of *M. ruber* M7 for all tested concentrations of adipic acid. The Δ*abct*31 colonial colors remained pale yellow up to the 9th day when compared with M. ruber M7 when the tested concentration of adipic acid was 1–3 mM used. The colonial color of *M. ruber* M7 strain also considerably changed to control the colonial growth of *M. ruber* M7. On the 12th day, the colonial color's profile of Δ*abct*31 strain changed for 2 and 3 mM concentration and the colonial colors turned to orange. As well as considering the colonial edges looked more irregular, the compacted growth of mycelium in the colonies could be observed when compared with the *M. ruber* M7, which were typically spread (Figure 13C).

So, in the following experiments, 7.5 mM sorbic acid, 1.5 mM acetic acid, and 3 mM adipic acid were used to check the *abct*31 gene response.

#### Expression Sensitivity of *abct*31 in *M. ruber* M7 Toward Weak Acids

**Figure 14:**
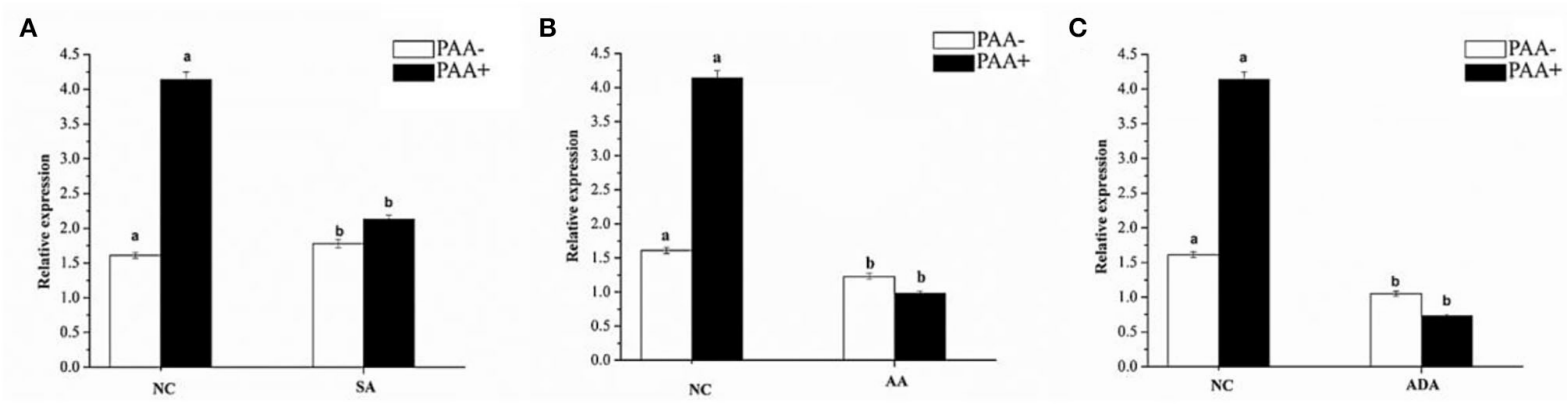
Expression of *abct*31 in *M. ruber* M7 to weak acid supplementation with or without PAA. NC was presented in the control group in which *M. ruber* M7 was cultivated on PDA without sorbic acid. SA presented the sorbic acid group in which *M. ruber* M7 was cultivated on a PDA with **(A)** Sorbic acid at 7.5 Mm; **(B)** Acetic acid at 1.5 Mm; **(C)** Adipic acid at 3 mM. Both groups were incubated at 28°C for 7d in the presence (black vertical bar) and absence (empty vertical bar) of PAA at 10 mM.

The effect of sorbic acid, adipic acid, and acetic acid at 7.5, 1.5, and 3 mM, respectively, on the expression level of the *abct*31 gene in *M. ruber* M7 with and without PAA at 10 mM has been investigated. The results are revealed in Figure 14.

From Figure 14, it has been observed that *abct*31 in *M. ruber* M7 exhibited a high transcription level with PAA when compared with the absence of PAA for all weak acids. Although the sorbic acid at 7.5 mM, acetic acid at 1.5 mM, and adipic acid at 3 mM significantly decreased the transcriptional level of *abct*31 in the absence of PAA, all the weak acids toxicity caused a highly significant (*p* < 0.01) decrease in the expression level of *abct*31 in the presence of PAA. Overall, sorbic acid at 7.5 mM causes the *abct*31 expression level more in the presence of PAA, whereas acetic acid at 1.5 mM and adipic acid at 3 mM cause more expression levels in the absence of PAA.

The findings of this work demonstrate that knocking out the ABC transporter *abct*31 in *M. ruber* M7 does not stop the formation of β-lactams, indicating that *abct*31 does not appear to play a direct part in this process. Nevertheless, when cells develop in +PAA, *abct*31 expression increases dramatically, indicating more functioning *abct*31 transport proteins in the cell. To see the effect on gene expression, we needed a high concentration of PAA in our PAA challenge assessments. Furthermore, when compared with its other β-lactam generating strains *P. chrysogenum*, the *abct*31 gene expression was higher in *M. ruber* M7 strains carrying only one copy of the penicillin biosynthetic gene cluster. Altogether, our findings indicate that *abct31* expression is influenced by the quantity of its inducer, PAA. When the *abct*31 gene was expressed, cells became more sensitive to PAA and sorbic acid, accompanied by a drop in *abct*31 transcript levels when the cells were subjected to these substances.

The side-chain precursor PAA, which is transferred from the cytosol to the peroxisomal matrix and is encoded by the *paaT* gene in *P. chrysogenum*, has recently been examined. It encodes a drug/H + antiporter with 12 TMSs, one of which is found in the membrane of peroxisomes as discovered using fluoresce targeted microscopy (Fernández-Aguado et al., 2013, 2014; Martín et al., 2013).

Many other weak acids, such as acetic and adipic acids, were shown to have no effect on the expression of *abct*31. As a result, they act as inducers through an as-yet-unidentified mechanism. It is possible that *abct*31 is not the only transporter engaged in exporting PAA. Expressions of the all-other residual ABC transporters were unaffected in the Δ*abct*31 strain cultured in the presence of PAA (unpublished data), indicating that no additional candidates for PAA export were discovered. Transporters from the major facilitator superfamily (MFS) may play a role in the residual low acid resistance potential. As a result of these findings, *abct*31 appears to play the same role as an ATP-dependent extrusion system for the weak acids to protect cells against these substances. These findings are backed by the theory that an ATP-dependent exporter is involved in the active secretion of the PAA (Xu et al., 2017).

According to our findings, it can be concluded that *M. ruber* M7 depends on at least two separate detoxifying mechanisms to deal with the PAA during fermentation. These mechanisms might be the conversion of PAA enzymatically into the PG and the active extrusion of PAA from the cell. However, the *abct*31 transporter is expressed in strains (deletion) with low penicillin G production rates, allowing a detoxifying process that also relies on the ATP-dependent extrusion of PAA.

## Conclusion

The outcomes of our study suggest that ABCT31 is the prime ABC transporter, primarily involved in this process of β-lactam synthesis. Additionally, based on these findings, it might be concluded that *M. ruber* depends upon two separate detoxifying mechanisms to deal with the higher PPA concentrations in the fermentation broth. These mechanisms could be the extrusion of PAA from the cell and then converting PAA into penicillin G by using enzymatic activity. Because of the residuals of PAA, the ABCT31 transporter is expressed and permits a detoxifying pathway. The detoxification mechanism also relies on the extrusion of PAA, which is ATP-dependent extrusion. Understanding the mechanisms governing the intake of these side-chain precursors aid in the clarification of the penicillin biosynthetic pathway. Still, it will also be beneficial economically for the β-lactams producers.

## Data Availability Statement

The data presented in the study are deposited in MetaboLights repository, accession number MTBLS4681.

## Author Contributions

RR has designed and carried out the present research work, conducted experiments, analyzed the data, and written the present manuscript. MV helped in doing the experiments and gave technical guidance. FC provided a place in the laboratory and gave access to the lab facilities for experimentation and funds for the present work. All authors contributed to the article and approved the submitted version.

## Funding

This work was supported by the Major Program of the National Natural Science Foundation of China (Nos. 31730068 and 31330059 to FC) and the National Key Research and Development Program of China (No. 2018YFD0400404 to FC).

## Conflict of Interest

The authors declare that the research was conducted in the absence of any commercial or financial relationships that could be construed as a potential conflict of interest.

## Publisher's Note

All claims expressed in this article are solely those of the authors and do not necessarily represent those of their affiliated organizations, or those of the publisher, the editors and the reviewers. Any product that may be evaluated in this article, or claim that may be made by its manufacturer, is not guaranteed or endorsed by the publisher.
